# CXCL13 contributes to chronic pain of a mouse model of CRPS-I via CXCR5-mediated NF-κB activation and pro-inflammatory cytokine production in spinal cord dorsal horn

**DOI:** 10.1186/s12974-023-02778-x

**Published:** 2023-05-08

**Authors:** Jie Wang, Chengyu Yin, Yushuang Pan, Yunqin Yang, Wei Li, Huadong Ni, Boyu Liu, Huimin Nie, Ruoyao Xu, Huina Wei, Yunwen Zhang, Yuanyuan Li, Qimiao Hu, Yan Tai, Xiaomei Shao, Jianqiao Fang, Boyi Liu

**Affiliations:** 1 Department of Neurobiology and Acupuncture Research, Key Laboratory of Acupuncture and Neurology of Zhejiang Province, The Third Clinical Medical College, Zhejiang Chinese Medical University, Hangzhou, 310053 China; 2 Department of Rehabilitation in Traditional Chinese Medicine, The Second Affiliated Hospital of Zhejiang University School of Medicine, Hangzhou, China; 3 NHC and CAMS Key Laboratory of Medical Neurobiology, MOE Frontier Science Center for Brain Research and Brain-Machine Integration, School of Brain Science and Brain Medicine, Zhejiang University, Hangzhou, China; 4 Department of Anesthesiology and Pain Research Center, The First Affiliated Hospital of Jiaxing University, Jiaxing, China; 5 Academy of Chinese Medical Sciences, Zhejiang Chinese Medical University, Hangzhou, China

**Keywords:** Neuroinflammation, Chemokine, Spinal cord, Pain, CXCL13, STAT3

## Abstract

**Background:**

Complex regional pain syndrome type-I (CRPS-I) causes excruciating pain that affect patients’ life quality. However, the mechanisms underlying CRPS-I are incompletely understood, which hampers the development of target specific therapeutics.

**Methods:**

The mouse chronic post-ischemic pain (CPIP) model was established to mimic CRPS-I. qPCR, Western blot, immunostaining, behavioral assay and pharmacological methods were used to study mechanisms underlying neuroinflammation and chronic pain in spinal cord dorsal horn (SCDH) of CPIP mice.

**Results:**

CPIP mice developed robust and long-lasting mechanical allodynia in bilateral hindpaws. The expression of inflammatory chemokine CXCL13 and its receptor CXCR5 was significantly upregulated in ipsilateral SCDH of CPIP mice. Immunostaining revealed CXCL13 and CXCR5 was predominantly expressed in spinal neurons. Neutralization of spinal CXCL13 or genetic deletion of *Cxcr5* (*Cxcr5*^−/−^) significantly reduced mechanical allodynia, as well as spinal glial cell overactivation and c-Fos activation in SCDH of CPIP mice. Mechanical pain causes affective disorder in CPIP mice, which was attenuated in *Cxcr5*^−/−^ mice. Phosphorylated STAT3 co-expressed with CXCL13 in SCDH neurons and contributed to CXCL13 upregulation and mechanical allodynia in CPIP mice. CXCR5 coupled with NF-κB signaling in SCDH neurons to trigger pro-inflammatory cytokine gene *Il6* upregulation, contributing to mechanical allodynia. Intrathecal CXCL13 injection produced mechanical allodynia via CXCR5-dependent NF-κB activation. Specific overexpression of CXCL13 in SCDH neurons is sufficient to induce persistent mechanical allodynia in naïve mice.

**Conclusions:**

These results demonstrated a previously unidentified role of CXCL13/CXCR5 signaling in mediating spinal neuroinflammation and mechanical pain in an animal model of CRPS-I. Our work suggests that targeting CXCL13/CXCR5 pathway may lead to novel therapeutic approaches for CRPS-I.

**Supplementary Information:**

The online version contains supplementary material available at 10.1186/s12974-023-02778-x.

## Introduction

Complex regional pain syndrome type-I (CRPS-I) is oftentimes triggered after an initial injury, including surgery, bone fracture, ischemia, etc. [1–3]. It usually develops in the extremities of the patients and may progress into other parts of body [2, 4]. It causes excruciating and chronic pain that dramatically affect patients’ life quality, both physically and mentally [1, 3]. The chronic pain and comorbidities also cause psychiatric disorders among CRPS patients, including anxiety, pain-related fear, depression, or even suicidal tendency [3, 5–8]. Unfortunately, current medications, including corticosteroids or non-steroidal anti-inflammatory drugs, do not exert satisfying therapeutic effects on CRPS-I patients, which makes it a challenging painful condition in clinic [4, 9]. Till now, the mechanism implicated in pain symptoms of CPRS-I is still far from being understood, thus limiting the possibility of finding effective therapeutics for the suffering patients.

Neuroinflammation is a critical process that contributes to the initiation and maintenance of chronic pain [10, 11]. Mounting evidence suggests that chemokines play an important role in mediating chronic pain through enhancing neuroinflammation [12, 13]. The chemokine CXCL13 was originally identified to be important for regulating B cell migration and lymphoid development via acting on CXCR5 receptor [14, 15]. Recent evidence demonstrated that CXCL13 and CXCR5 are also expressed in sensory nerve system, including peripheral sensory ganglion and spinal cord dorsal horn (SCDH), and contribute to chronic pain [16–20]. For example, CXCL13 expression is robustly increased in SCDH neurons after spinal nerve ligation and activates astrocytes via neuron–glia crosstalk to promote neuroinflammation, which subsequently causes neuropathic pain [16]. In addition, CXCL13 is upregulated in an animal model of inflammatory pain and acts on CXCR5 on dorsal root ganglion (DRG) neurons to promote Nav1.8 channel expression and contributes to inflammatory pain [18]. These studies showed that CXCL13/CXCR5 signaling contributes to neuropathic or inflammatory pain through sensitization of neurons, activation of glial cells and promoting neuroinflammation. But it still remains unknown whether and how CXCL13/CXCR5 signaling may participate in the pathogenesis of CRPS-I.

Spinal cord dorsal horn (SCDH) is an important place for pain signal convergence, integration, relay and central sensitization. We and others found that astrocytes and microglia cells were overactivated in ipsilateral SCDH of an animal model of CRPS-I [21–23]. Our work further identified that inflammatory response were the predominant biological process in SCDH of CRPS-I model animals [24]. Given the fact that neuroinflammation plays a critical role in mediating chronic pain, here we explored the potential pro-inflammatory cytokines or chemokines in SCDH of a mouse CRPS-I model that might participate in pain mechanisms. We found that CXCL13 was among the highest upregulated chemokines by bioinformatics and protein validation. We further demonstrated that CXCL13 was produced from neurons in SCDH via a STAT3-dependent mechanism. CXCL13 acts via CXCR5 to initiate NF-κB signaling activation in spinal neurons, which triggered pro-inflammatory cytokine production, glial cell overactivation and contribute to chronic pain. Our work may provide novel insights into identifying pain relieving targets for CRPS-I.

## Methods and materials

### Animals

Wild-type C57BL/6J (male and female, 6–8 weeks old) were purchased from Shanghai Laboratory Animal Center, Chinese Academy of Sciences. *Cxcr5* global knockout (*Cxcr5*^−/−^) mice in the background of C57BL/6J were kindly provided by Prof. Yongjing Gao at Nantong University, China. All animals were housed in the Laboratory Animal Center of Zhejiang Chinese Medical University under standard environmental conditions (12 h light–dark cycle and 24 ± 2 °C). Animals were randomly allocated. Five mice were housed per cage with access to food and water freely. Animals were given a minimum of 1 week to adapt to new environment before experiment. All animal care and experimental studies were approved by the Laboratory Animal Management and Welfare Ethical Review Committee of Zhejiang Chinese Medical University.

### CPIP mouse model establishment

Chronic post-ischemia pain (CPIP) was generated following exposure to prolonged hind paw ischemia reperfusion injury [25]. Mice were anesthetized over a 3-h period with an initial bolus (50 mg/kg, i.p.) and supplements (20 mg/kg, i.p.) of sodium pentobarbital when required. After induction of anesthesia, the mouse right hind paw was inserted into a 200-μL PCR tube (with the snap-cap cut off before use). Then a Nitrile 70 Durometer O-ring with a 2 mm internal diameter was slide to the larger side of the tube (with the snap-cap cut off before use) and gradually slide to the mouse’s right ankle joint. The tube was then removed. The placement of the O-ring on the ankle joint lasted for 3 h, as was described with the larger O-rings in rats [26, 27]. The O-ring was cut off 3 h later for reperfusion. Sham mice were anesthetized as CPIP mice but with no O-ring placed on the ankle.

### Determination of mechanical allodynia and hindpaw swelling

Mice were habituated to the testing environment daily for at least 2 days before baseline testing. Animals were individually placed in transparent Plexiglas chambers on an elevated mesh floor and were habituated for 30 min before the test. The mechanical hyperalgesia was determined using a series of von Frey filaments (UGO Basile, Italy) applied perpendicularly to the midplantar surface of the hind paws, with sufficient force to bend the filament slightly for 3–5 s according to methods we previously described [28]. The “Up-Down” testing paradigm was used to determine the threshold, and the 50% paw withdrawal threshold (PWT) was calculated by the non-parametric Dixon test [29]. A digital caliper was used to measure the swelling observed as an increase in hind paw diameter. The difference between the basal value and the test value was calculated as in our previous study [24]. Changes in paw diameter were shown as % increase in paw diameter and calculated as follows: % increase in paw diameter = (D_after_ − D_before_)/D_before_. Each mouse was measured 3 times, and the mean value was calculated.

### Determination of cold allodynia

Cold allodynia was determined by acetone-induced evaporative cooling test as described in our previous work [30]. Briefly, the mice were habituated to the testing environment for 30 min before test. A drop of 20 μl acetone was sprayed to the plantar surface of the hindpaw. The nocifensive behavior, including licking, flicking and biting the hindpaw was recorded with a video camera located beneath for 1 min and calculated. Cold allodynia was judged as an increase in nociceptive time observed after exposure to acetone.

### Real-time place escape/avoidance (PEA) test

Animals were subjected to the classical 2-chamber conditioned place aversion (CPA) test for PEA. The size of each chamber was 40 × 28 × 32 cm. Mice were habituated to the testing environment for 1 day. We placed the chamber on the mesh floor. The chamber was made with plastic plates that had distinct pattern from one another. This test was divided into 3 stages. In the first 10 min, the mouse was allowed to explore both chambers without mechanical stimulation (pre-stimulation). Usually, after exploration, the mouse showed a small preference for one of the two chambers. In the second 10 min, 0.4 g von Frey hair was applied to the right (ipsilateral) hind paw whenever the mouse entered or stayed in the preferred chamber (stimulation). A cut-off of 2 s was set in each stimulation and the interval of stimulations was 10 s. In the last 10 min, the mouse could freely explore both chambers (post-stimulation). ANY-Maze system (Stoelting, USA) was used to record and analyze the mouse’s movement and the time stayed in each chamber. All behavior tests were tested blindly by the experimenter.

### qPCR

The ipsilateral lumber SCDH was collected and was preserved in RNA*later* solution (Thermo Fisher, Carlsbad, USA). We used Trizol reagent (Thermo Fisher, USA) to extract total RNA and Prime ScriptTM RT reagent Kit (Takara Bio Inc., China) to reversely transcribe the total RNA. The concentration and purity of each sample was detected by Nanodrop Spectrophotometer (Nanodrop, USA). qPCR was performed by CFX96 Real-Time System (Bio-Rad, USA) using the Fast Start Universal SYBR Green Master kit (Takara Bio Inc, China). Each reaction was performed in triplicates and normalized to *β-actin* gene expression. ΔΔCT method was used to determine the relative expression [31]. Detailed primer sequences are listed in Additional file 10: Table S1.

### Western blot

Animals were killed after behavioral testing on days 7 or days 14. Ipsilateral lumber SCDH and DRG (L3–L5) were quickly obtained. Tissues were homogenized in RIPA buffer containing proteinase inhibitor and phosphatase inhibitor and the supernatant was collected after centrifuging. The protein concentrations were determined by BCA assay (Thermo Fisher, USA) with 20 μg protein in each lane. Protein for each lane was loaded and separated by 8–15% SDS-PAGE and transferred to PVDF membranes. The blots were blocked with 5% non-fat milk or bovine serum albumin (BSA) at room temperature for 1 h, probed with primary antibodies at 4 °C overnight, and incubated with HRP-coupled secondary antibodies. In case where the protein bands for the protein of interest and control (β-actin) are the same size, the blot would be stripped of original 1st antibody staining and re-probed for subsequent experiment. The 1st antibodies used are as follows: CXCL13 (#PA5-28827, 1:1000, rabbit polyclonal, Thermo Fisher), CXCR5 (#254415, 1:2000, rabbit monoclonal, Abcam), STAT3 (#9139, 1:1000, mouse monoclonal, CST), pSTAT3 (#9145, 1:2000, rabbit monoclonal, CST), Ace-STAT3 (#2523, 1:1000, rabbit polyclonal, CST), NF-κB (#8242, 1:2000, rabbit monoclonal, CST), pNF-κB (#3033, 1:2000, rabbit monoclonal, CST), pERK (#4370, 1:2000, rabbit monoclonal, CST). β-actin (#20272, 1:5000, mouse monoclonal, Abcam) was used as reference loading control. Immunoreactive bands were detected with enhanced chemiluminescent substrate (Bio-Rad, USA) and visualized using Image Quant LAS 4000 (GE, USA). The results of protein expression are normalized to β-actin and analyzed using ImageJ software (USA).

### Immunofluorescent staining

Mice were deeply anaesthetized with isoflurane and perfused transcardially with 4 °C saline followed by 4% fresh paraformaldehyde. Then lumber spinal cord was harvested. The tissues were post-fixed in 4% paraformaldehyde in 0.1 M PBS for 4–6 h (4 °C) before transferring to 15% for 24 h and 30% sucrose for 72 h for dehydration. Spinal cord was serially cut into 20 μm thickness section on a frozen microtome (Thermo NX50, USA) and processed for immunofluorescence. The sections were blocked with 5% donkey serum in TBST (with 0.3% Triton X-100, blocking buffer) for 1 h at 37 °C, then incubated with the following primary antibodies at 4℃ overnight: CXCL13 antibody (#PA5-28827 1:200, rabbit polyclonal, Thermo Fisher), CXCR5 antibody (#254415, 1:200, rabbit monoclonal, Abcam), pSTAT3 antibody (#9145, 1:200, rabbit monoclonal, CST & #4113, 1:200, mouse monoclonal, CST), pNF-κB antibody (#sc-136548, 1:200, mouse monoclonal, SCBT), c-Fos antibody (#2250, 1:200, rabbit monoclonal, CST), NeuN antibody (#104224, 1:300, mouse monoclonal, Abcam), GFAP antibody (#3670s, 1:200, mouse monoclonal, CST), Iba1 antibody (#011-27991, 1:500, goat polyclonal, Wako). The sections were then incubated 1 h at 37 °C with corresponding secondary antibodies, including #Ab150061 Donkey Anti-Rabbit IgG H&L (Alexa Fluor^®^ 488), #Ab150064 Donkey Anti-Rabbit IgG H&L (Alexa Fluor^®^ 594), #Ab150111 Donkey Anti-Mouse IgG H&L (Alexa Fluor^®^ 647), #Ab150129 Donkey Anti-Goat IgG H&L (Alexa Fluor^®^ 488) (Abcam) and #705-605-147 Donkey Anti-Goat IgG H&L (Alexa Fluor^®^ 647) (Jackson ImmunoResearch). Fluorescence images were captured by Nikon A1R laser scanning confocal microscope (Nikon, Japan). For image quantification, uniform microscope settings were maintained throughout all image capture sessions. Three images were randomly selected per mouse tissue, then averaged, and compared according to methods described in our previous studies [32]. A GFAP or Iba-1-positive cell was defined as having 2 characters: 1. stained positive for GFAP or Iba-1; 2: having a DAPI labeled nucleus. The counting was performed by an experimenter blinded to the grouping.

### Drug administration

CXCL13 (100 ng/10 μL) and its neutralizing antibody (20 ng/10 μL) were purchased from R&D systems (USA). S3I-201 (STAT3 inhibitor, 10 μg/10 μL) was purchased from Santa Cruz Biotechnology (USA). Ammonium pyrrolidinedithiocarbamate (PDTC, NF-κB inhibitor, 300 μg/5 μL) was purchased from Merck (USA). CXCL13 neutralizing antibody (20 ng/10 μl) was purchased from R&D Systems (USA). IL-6 neutralizing antibody (10 ng/10 μl) was purchased from InvivoGen (USA). Isotype control IgG was purchased from Abcam (UK). The doses of the reagents were chosen according to previous studies [13, 16, 33–35]. Reagents were dissolved as following: S3I-201 in 2% DMSO + 98% PBS, PDTC in 20% DMSO + 80% PBS, etanercept in 10% DMSO + 90% PBS, CXCL13 in 0.1% BSA and IL-6 neutralizing antibody in 100% PBS. The drugs and their corresponding vehicle were administrated intrathecally (i.t.) 1 h before behavioral test. Intrathecal injection was performed by a lumbar puncture to deliver to cerebral spinal fluid under isoflurane anesthesia as we previously described [36].

### Statistical analysis

Results are expressed as means ± SEM. Student’s *t* test (two-tailed) was used for comparisons between two groups. One- or two-way ANOVA followed by Tukey’s post hoc test was used for comparison among 3 or more groups. ANOVA with repeated measures were taken wherever needed. Comparison is considered significantly different if *p* < 0.05.

## Results

### The CRPS-I mouse model developed persistent mechanical allodynia and showed CXC13/CXCR5 upregulation in ipsilateral spinal cord dorsal horn

**Fig. 1:**
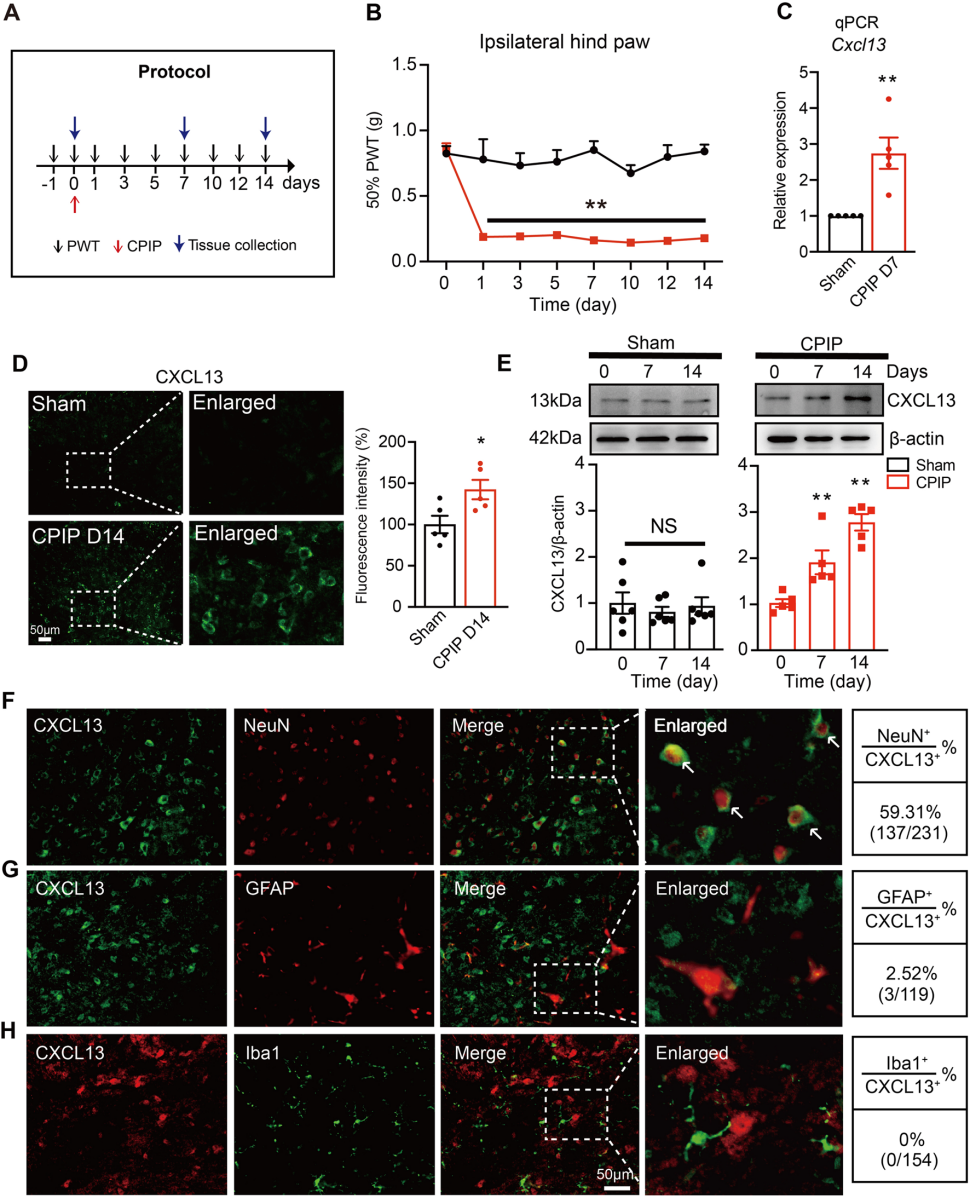
CRPS-I mouse model showed persistent mechanical allodynia, accompanied with CXCL13 upregulation in ipsilateral spinal cord dorsal horn. **A** Protocol for the experiments. **B** 50% paw withdrawal threshold (PWT) measured in ipsilateral hindpaw before and after CPIP model establishment. **C** qPCR of *Cxcl13* gene expression in ipsilateral SCDH of sham and CPIP model mice on Day 7. **D** Immunostaining showing CXCL13 expression in ipsilateral SCDH of CPIP mice on Day 14. Right panel: summary of normalized fluorescence intensity of CXCL13. Values were normalized with sham group. **E** Western blot showing CXCL13 expression in sham (left panel) and CPIP model group (right panel) on Day 0, 7 and 14. The upper panels show representative immunoblot images and the lower panels show pooled data. *n* = 5–6 mice/group. **F**–**H** Double immunostainings of CXCL13 with NeuN (neuron marker, **F**), GFAP (astrocyte marker, **G**) and Iba-1 (microglia marker, **H**). Quantification of cellular distribution was shown on the right (pooled from 4 to 5 mice/group). Scale bar indicates 50 μm. Two-way ANOVA with repeated measures followed with Tukey post hoc test was used for comparisons in **A**. Student’s *t* test was used for comparisons in **C**, **D**. One-way ANOVA followed with Tukey post hoc test was used for comparisons in **E**. **p < 0.01 vs. sham or CPIP D0 group

We first established the CPIP model to replicate human CRPS-I in mice according to previous literatures [37, 38]. After the placement of the O-ring on the ankle region of mice, ipsilateral hindpaws developed remarkable swelling and cyanosis, which were signs of tissue ischemia and hypoxia (Additional file 1**:** Fig. S1A). Ten minutes after reperfusion, obvious paw swelling were observed in ipsilateral hindpaw (Additional file 1: Fig. S1A). The swelling in ipsilateral side gradually diminished 3 days later (Additional file 1: Fig. S1B). The ipsilateral hindpaw developed a significant reduction in 50% paw withdrawal threshold, a sign of mechanical allodynia. The mechanical allodynia lasted over 14 days (Fig. 1A, B). Although no swelling ever occurred, contralateral hindpaw developed obvious and persistent mechanical allodynia similarly to ipsilateral hindpaw, which was a typical sign of mirror-image pain (MIP) [39] (Additional file 1: Fig. S1C, D&E). The above manifestations were all consistent with previous study [38], demonstrating the successful establishment of CRPS-I animal model.

We aimed to explore potential pain mechanisms in spinal cord dorsal horn (SCDH) of CPIP mice. We recently performed RNA-Seq on ipsilateral spinal cord to explore potential pain mechanisms of a rat model of CPIP 7 days after model establishment. Our study indicated that inflammatory responses were the predominant biological process in SCDH [24]. We then set to screen all inflammation-related cytokines or chemokines within the RNA-Seq dataset and found that *Cxcl13* gene was among the most upregulated genes in CPIP model rats. We next validated *Cxcl13* gene expression in SCDH of CPIP model mice. qPCR indicated that gene expression of *Cxcl13* were significantly increased in ipsilateral SCDH of CPIP group than sham group (Fig. 1C). Immunostaining showed that CXCL13 immunoreactivities (IR) were significantly increased in ipsilateral SCDH of CPIP group compared with sham group (Fig. 1D). Western blot further demonstrated that protein levels of CXCL13 were significantly increased in spinal cord of CPIP group vs. sham group on Day 7 and Day 14 (Fig. 1E). We examined cellular distributions of CXCL13 in SCDH of CPIP mice. We performed immunostaining of CXCL13 and co-labeled them with specific markers for different types of cells in spinal cord. These markers include GFAP for astrocytes, NeuN for neurons and Iba-1 for microglia. We found that CXCL13 exhibited co-expression exclusively with NeuN, but scarcely with GFAP or Iba-1 (Fig. 1F–H).

**Fig. 2:**
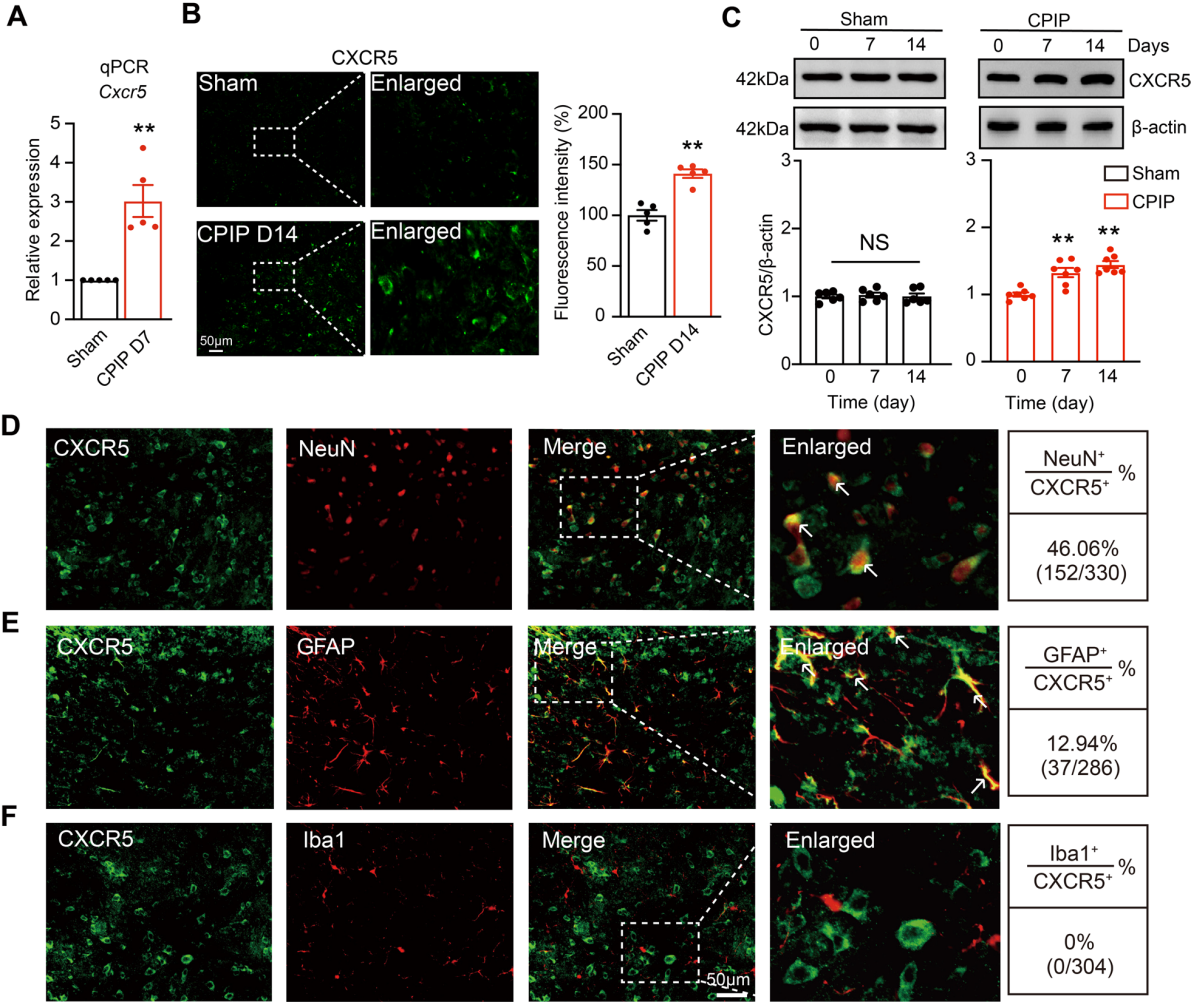
The expression of CXCR5 is increased in ipsilateral spinal cord dorsal horn of CPIP model mice. **A** qPCR analysis of gene expression level of *Cxcr5* in ipsilateral SCDH of sham and CPIP model mice on Day 7. **p < 0.01 vs. sham. **B** Immunostaining showing CXCR5 expression in ipsilateral SCDH of CPIP mice on Day 14. Right panel: summary of normalized fluorescence intensity of CXCR5. All values were normalized with sham group. **p < 0.01 vs. sham. Scale bar indicates 50 μm. *n* = 5 mice/group. **C** Western blot showing CXCR5 expression in sham (left) and CPIP model (right) group on Day 0, 7 and 14. *n* = 5–7 mice/group. **p < 0.01 vs. CPIP D 0 group. **D**–**F** Double immunostainings of CXCR5 with NeuN (**D**), GFAP (**E**) and Iba-1 (**F**). Quantification of cellular distribution was shown on the right (pooled from 4 to 5 mice/group). Scale bar indicates 50 μm. Student’s *t* test was used for comparisons in **A**, **B**. One-way ANOVA followed with Tukey post hoc test was used for comparisons in **C**

We next studied the expression of CXCL13 receptor CXCR5. qPCR showed that *Cxcr5* gene expression was significantly increased in ipsilateral SCDH of CPIP group (Fig. 2A). Immunostaining showed that CXCR5 IR were significantly increased in ipsilateral SCDH of CPIP group vs. sham group (Fig. 2B). Western blot further demonstrated that protein levels of CXCR5 were significantly increased in ipsilateral SCDH of CPIP group vs. sham group on Day 7 and Day 14 (Fig. 2C). Moreover, CXCR5 showed co-expression predominantly with NeuN and to a much less extent with GFAP, but barely with Iba-1 in ipsilateral SCDH of CPIP model mice (Fig. 2D–F). In contrast, CXCL13 or CXCR5 expression was not significantly changed in contralateral SCDH of CPIP model mice (Additional file 2: Fig. S2A-D). The specificity of the CXCR5 antibody was further validated using *Cxcr5* global knockout (*Cxcr5*^−/−^) mice. The IR of CXCR5 in SCDH was largely abolished in *Cxcr5*^−/−^ mice, demonstrating the specificity of the CXCR5 antibody we used (Additional file 3: Fig. S3A&B). Upregulation of CXCL13/CXCR5 in DRG also makes contribution to chronic pain [18]. We evaluated CXCL13/CXCR5 expression in ipsilateral L3–L5 segments of DRG from CPIP and sham group on Day 7 and 14. Immunostaining or Western blot did not identify any obvious increase in CXCL13/CXCR5 expression in DRG (Additional file 4: Fig. S4A-F). These data demonstrate the expressions of CXCL13 and CXCR5 are significantly increased in ipsilateral SCDH of CPIP mice.

### Spinal CXCL13 and CXCR5 contribute to pain mechanisms of CPIP mice

**Fig. 3:**
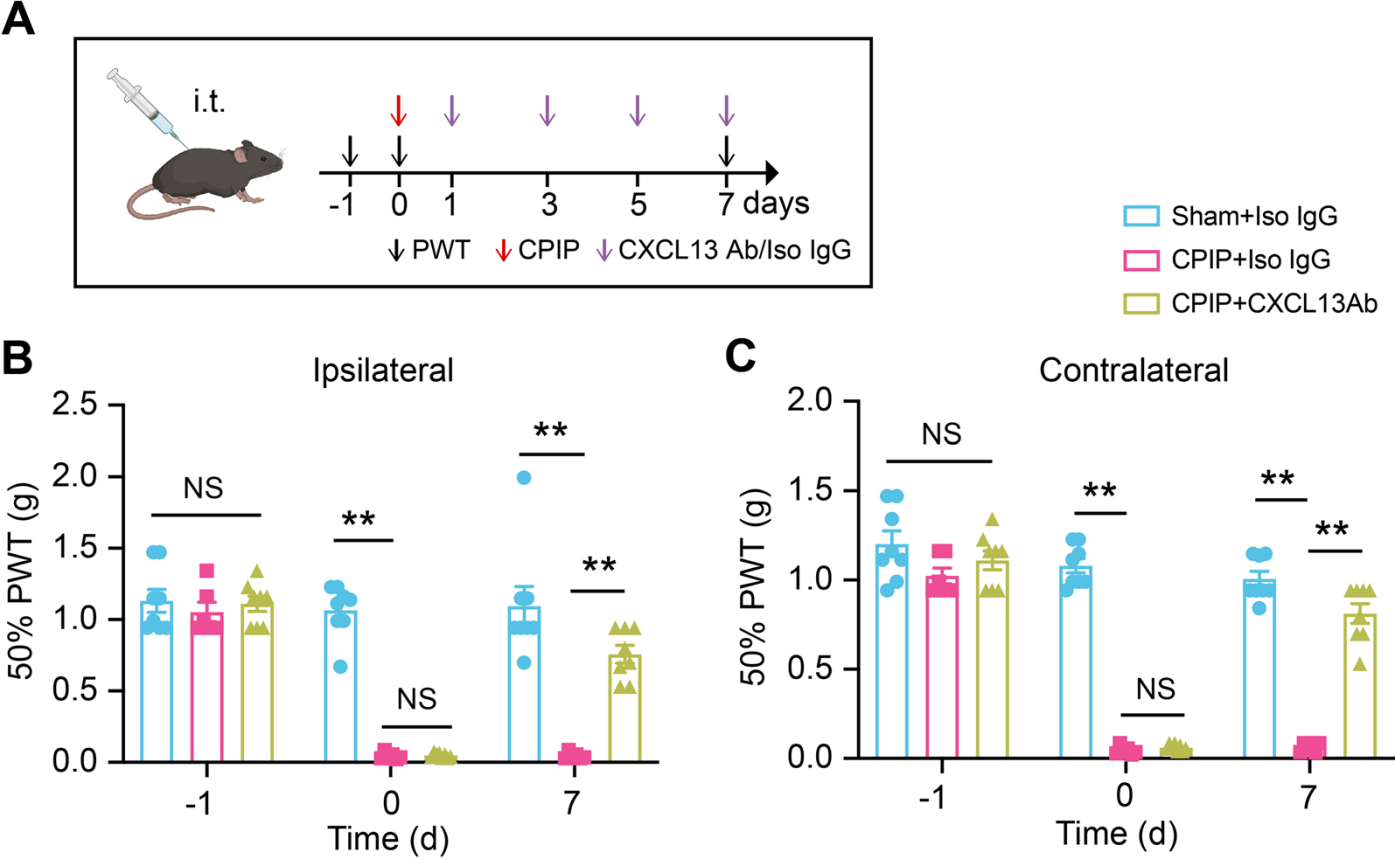
Spinal blocking CXCL13 attenuates mechanical allodynia of CPIP model mice. **A** Experimental protocol showing the time points for CPIP model establishment, intrathecal injections of CXCL13 neutralizing antibody (Ab, 20 ng/10 μl) or corresponding isotype control IgG (Iso IgG) and behavioral test. **B** 50% PWT in ipsilateral hindpaw after intrathecal CXCL13 Ab or Iso IgG treatment in CPIP and sham group. **C** 50% PWT in contralateral hindpaw. Two-way ANOVA with repeated measures followed by Tukey’s post hoc test was used for comparisons. *n* = 8 mice/group. **p < 0.01. NS: no significance

We studied whether CXCL13 was involved in mechanical allodynia of CPIP mice. We blocked CXCL13 using a neutralizing antibody. CXCL13 neutralizing antibody or corresponding isotype control IgG was applied via intrathecal injection at time points indicated in Fig. 3A. Neutralizing CXCL13 in spinal cord significantly ameliorated bilateral mechanical allodynia of CPIP mice compared with isotype control IgG (Fig. 3B, C).

**Fig. 4:**
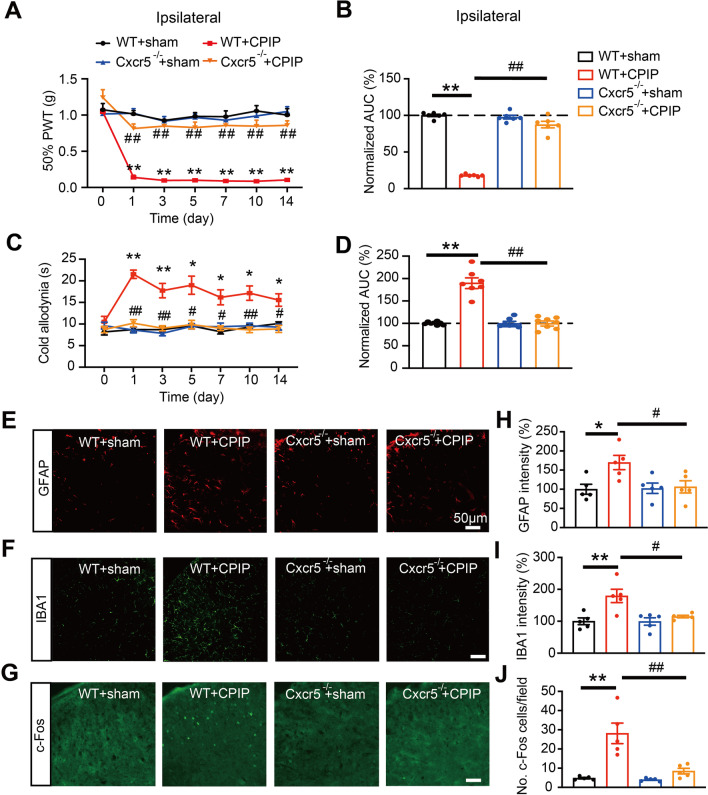
CXCR5 is essential for mediating mechanical/cold allodynia as well as c-Fos and glial cell overactivation in ipsilateral SCDH of CPIP mice. **A** Time course showing 50% PWT changes in ipsilateral hindpaws of WT and *Cxcr5*^−/−^ mice after CPIP model establishment. **B** Summary of normalized AUC of curves in **A**. **C** Time course showing cold allodynia behaviors in ipsilateral hindpaws of WT and *Cxcr5*^−/−^ mice upon acetone application. **D** Summary of normalized AUC of curves in **C**. **E**–**G** Immunostaining of GFAP (**E**), Iba-1 (**F**) and c-Fos (**G**) in ipsilateral SCDH from WT + sham, WT + CPIP, *Cxcr5*^−/−^ + sham and *Cxcr5*^−/−^ + CPIP groups. **H**–**J** Summary of GFAP (**H**) & Iba-1 (**I**) fluorescence intensity and c-Fos (**J**) positively stained cell number. *n* = 5–6 mice/group. *p < 0.05, **p < 0.01 vs. WT + sham group; ^#^*p* < 0.05, ^##^*p* < 0.01 vs. WT + CPIP group. Scale bar indicates 50 μm. Two-way ANOVA with repeated measures followed by Tukey’s post hoc test was used for comparisons in **A**, **C**. One-way ANOVA followed by Tukey’s post hoc test was used for comparisons in others

We then used *Cxcr5*^−/−^ mice to test the involvement of CXCR5 in CPIP-induced wildtype (WT) model mice (WT + CPIP). As shown in Fig. 4A, CPIP-induced WT model mice developed persistent mechanical allodynia in ipsilateral hindpaws, whereas mechanical allodynia was remarkably attenuated in *Cxcr5*^−/−^ mice at all time points we examined. Area under the curve (AUC) analysis further indicated an accumulated attenuation of mechanical allodynia in *Cxcr5*^−/−^ mice compared with WT mice (Fig. 4B). CPIP-induced WT model mice also developed cold allodynia in ipsilateral hindpaw, a sign consistent with previous study [37]. *Cxcr5*^−/−^ mice showed significantly attenuated cold allodynia compared with WT mice in CPIP condition (Fig. 4C, D). Since we found CXCR5 expression was predominantly located in neurons and astrocytes in SCDH, we then explored whether CXCR5 might contribute to neuronal and glial activation in SCDH in CPIP condition. Immunostaining revealed that the basal IR for GFAP, Iba1 as well as the number of c-Fos positively stained cells in ipsilateral SCDH were similar in uninjured WT and *Cxcr5*^−/−^ mice (WT + sham vs. *Cxcr5*^−/−^ + sham, Fig. 4E–G). The IR for GFAP and Iba-1 and c-Fos positively stained cell number were all significantly increased in ipsilateral SCDH of WT mice on Day 14 compared with sham group after model establishment (WT + CPIP vs. WT + sham). Of note, a number of astrocytes showed enlarged cell bodies and thickening of process, whereas microglia showed hypertrophy of the cell body and branching process increases in CPIP model mice, an indication of cell activation (Additional file 5: Fig. S5A&B). These increases in IR for GFAP and Iba-1 and c-Fos positively stained cell number were significantly attenuated in CPIP + *Cxcr5*^−/−^ group on Day 14 (Fig. 4E–J). Open field test indicated similar travel distances in central zone among WT and *Cxcr5*^−/−^ mice, ruling out possible deficits in locomotor activity (Additional file 6: Fig. S6A-D).

CPIP mice also developed mechanical allodynia in contralateral hindpaw, which was significantly attenuated in *Cxcr5*^−/−^ mice (Additional file 7: Fig. S7A&B). The IR for GFAP and Iba-1 and c-Fos positively stained cell number were significantly increased in contralateral SCDH of WT + CPIP group on Day 14 vs. WT + sham group, whereas CPIP + *Cxcr5*^−/−^ group showed much attenuated above increases in contralateral side (Additional file 7: Fig. S7C-H).

It has become more and more acknowledged that sex plays an important role in pain mechanisms. The above experiments were all performed in male mice. In order to examine whether CXCR5 may drive mechanical pain through a sex-dependent manner, we tested female mice in our following study. Similar to male mice, female WT mice developed obvious and persistent mechanical allodynia in bilateral hindpaws after CPIP model establishment (Additional file 8: Fig. S8A-D). Female *Cxcr5*^−/−^ mice showed much reduced bilateral mechanical allodynia compared with female WT mice under CPIP condition (Additional file 8: Fig. S8A-D). Besides, the IR for GFAP and Iba1, as well as c-Fos positively stained cell number, were all increased in ipsilateral SCDH of female WT mice on Day 14, whereas these increases were all significantly reduced in female *Cxcr5*^−/−^ mice (Additional file 8: Fig. S8E-J). This suggests that CXCR5 is responsible for mechanical allodynia of CPIP model mice of both sexes.

**Fig. 5:**
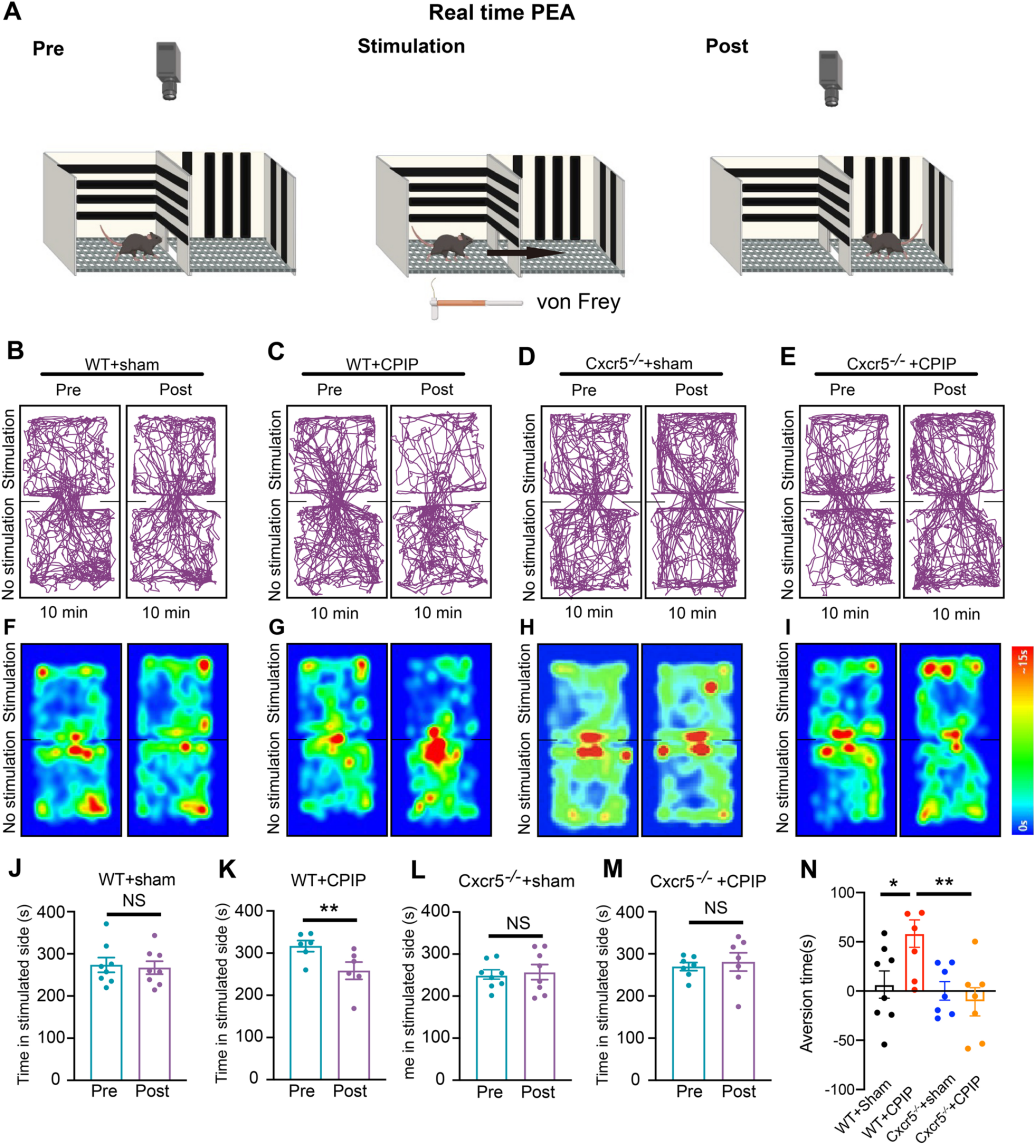
*Cxcr5*^−/−^ mice showed deficits in real-time place escape/avoidance behavior induced by CPIP. **A** Schematic picture showing real-time PEA test performed on Day14 after CPIP model establishment. During pretest phase, the mouse is placed in a two-chamber box and allowed to explore both chambers without stimulation. During stimulation phase, a von Frey hair is used to stimulate the ipsilateral hindpaw whenever the mouse enters or stays in the preferred chamber. During the posttest phase, the mouse can freely explore the chambers without stimulation. **B**–**E** Representative pictures showing mouse moving traces of WT + Sham, WT + CPIP, *Cxcr5*^−/−^ + Sham and *Cxcr5*^−/−^ + CPIP groups during pretest and posttest phases (10 min for each). **F**–**I** Corresponding heatmap illustrations of the moving traces in accordance with **B**–**E**. **J**–**M** Comparison of the dwelling time in stimulated chamber of WT + Sham (**J**), WT + CPIP (**K**), *Cxcr5*^−/−^ + Sham (**L**) and *Cxcr5*^−/−^ + CPIP (**M**) groups of mice before and after mechanical stimulation. **N** Calculated aversion time of all 4 groups of mice. *n* = 6–8 mice/group. *p < 0.05, **p < 0.01. NS: no significance. Student’s *t* test was used for comparisons in panels **J**–**M**. One-way ANOVA followed by Tukey’s post hoc test was used for comparisons in **N**

CRPS-I patients usually exhibit emotional distress and affective disorders due to chronic pain [40]. We explored whether CPIP model mice developed affective responses as well. We used a modified real-time place escape/avoidance (PEA) test with mechanical stimulation for the evaluation [41] (Fig. 5A). We found that WT + CPIP group mice spent significantly less time on mechanically stimulated side and exhibited aversion behavior during post-stimulation phase compared with WT + sham group mice 14 days after model establishment. In contrast, *Cxcr5*^−/−^ + CPIP group mice did not develop any obvious signs of aversion upon mechanical stimulation compared with WT + CPIP group mice (Fig. 5B–N). In together, these results demonstrated that CXCL13/CXCR5 signaling contributes to mechanical and cold allodynia as well as affective response of CPIP mice.

### STAT3 phosphorylation contributes to CXCL13 upregulation in SCDH of CPIP model mice

**Fig. 6:**
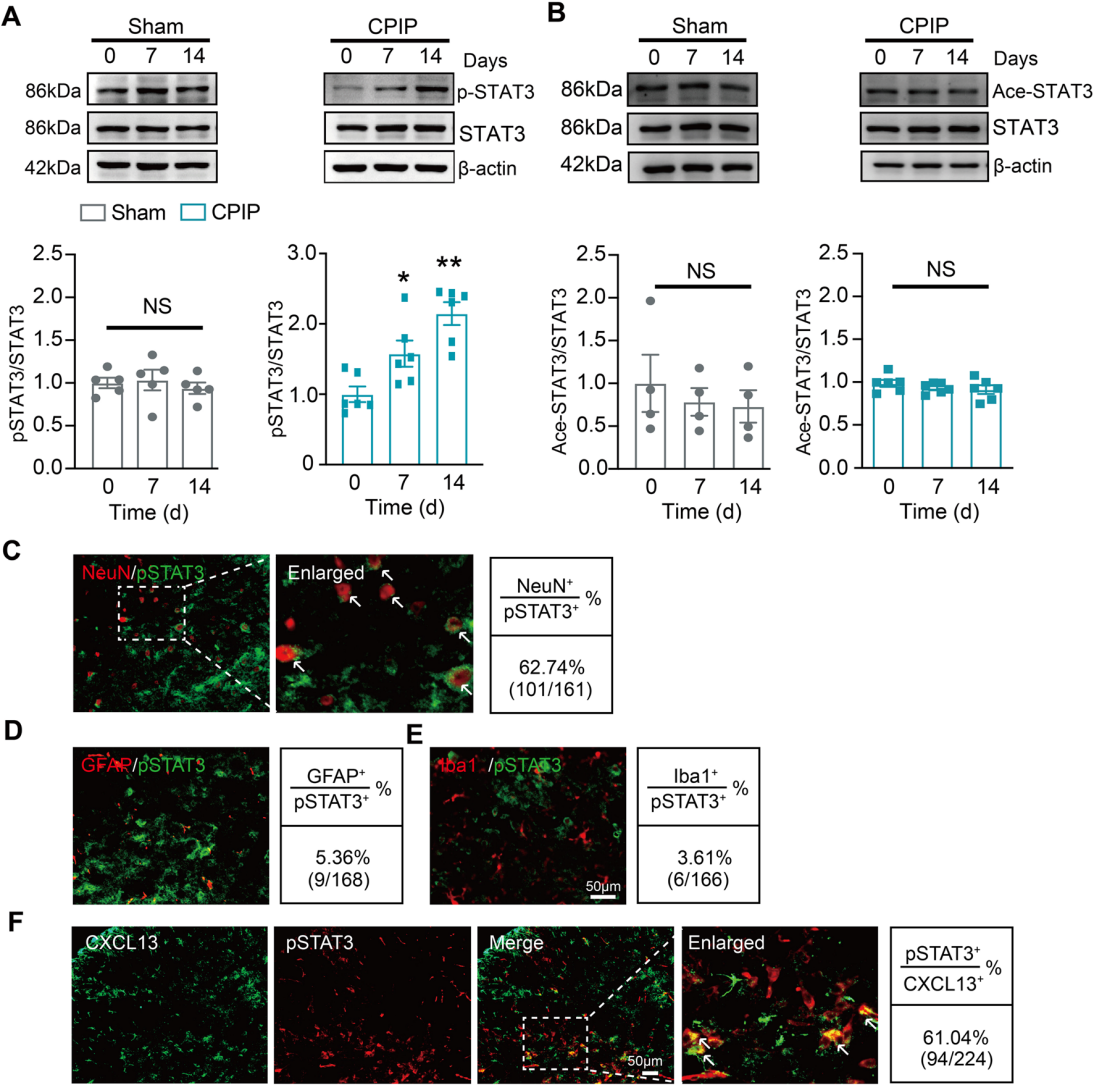
STAT3 is activated and co-localized with CXCL13 in ipsilateral SCDH of CPIP mice. **A** Western blot showing expression of p-STAT3 and STAT3 in sham (left) and CPIP model (right) mice on Day 0, 7 and 14. Upper panels indicated representative blot images and lower panel indicate pooled data. **B** Western blot showing expression of acetylated-STAT3 (Ace-Stat3). *n* = 4–6 mice/group. **C**–**E** Double immunostaining of p-STAT3 with NeuN (**C**), GFAP (**D**) and Iba-1 (**E**) in ipsilateral SCDH of CPIP mice. White arrows indicate p-STAT3 and NeuN co-labeled cells. **F** Double immunostaining of p-STAT3 with CXCL13 in ipsilateral SCDH of CPIP mice. White arrows indicate p-STAT3 and CXCL13 co-labeled cells. Quantification of cellular distribution was shown on the right (pooled from 4 to 5 mice/group). *p < 0.05, **p < 0.01 vs. Day 0. NS: no significance. Scale bar indicates 50 μm. One-way ANOVA followed by Tukey’s post hoc test was used for comparisons in **A**, **B**

STAT3 serves as an important transcription factor to modulate chemokine or cytokine expression in nerve system and contributes to chronic pain [42, 43]. We asked whether STAT3 was involved in CXCL13 expression regulation in SCDH of CPIP mice. Western blot showed that phosphorylated-STAT3 (p-STAT3) level, but not total STAT3 level, was significantly enhanced on Day 7 and 14 in ipsilateral SCDH of CPIP mice vs. sham mice (Fig. 6A). We further checked whether acetylation of STAT3, a critical process for modulating STAT3 signaling, also took place in SCDH of CPIP mice [44]. As shown in Fig. 6B, the acetylated STAT3 (ace-STAT3) level remained unchanged after model establishment. Immunostaining indicated that p-STAT3 expression was exclusively co-expressed with NeuN-labeled neurons, but rarely with GFAP-labeled astrocytes or Iba1-labeled microglia (Fig. 6C–E). Immunostaining further unraveled that p-STAT3 expression co-localized with CXCL13 positively labeled cells in ipsilateral SCDH of CPIP mice (Fig. 6F). Thus, the co-localization of p-STAT3 with CXCL13 in SCDH neurons provides the possibility for p-STAT3 to modulate CXCL13 expression.

**Fig. 7:**
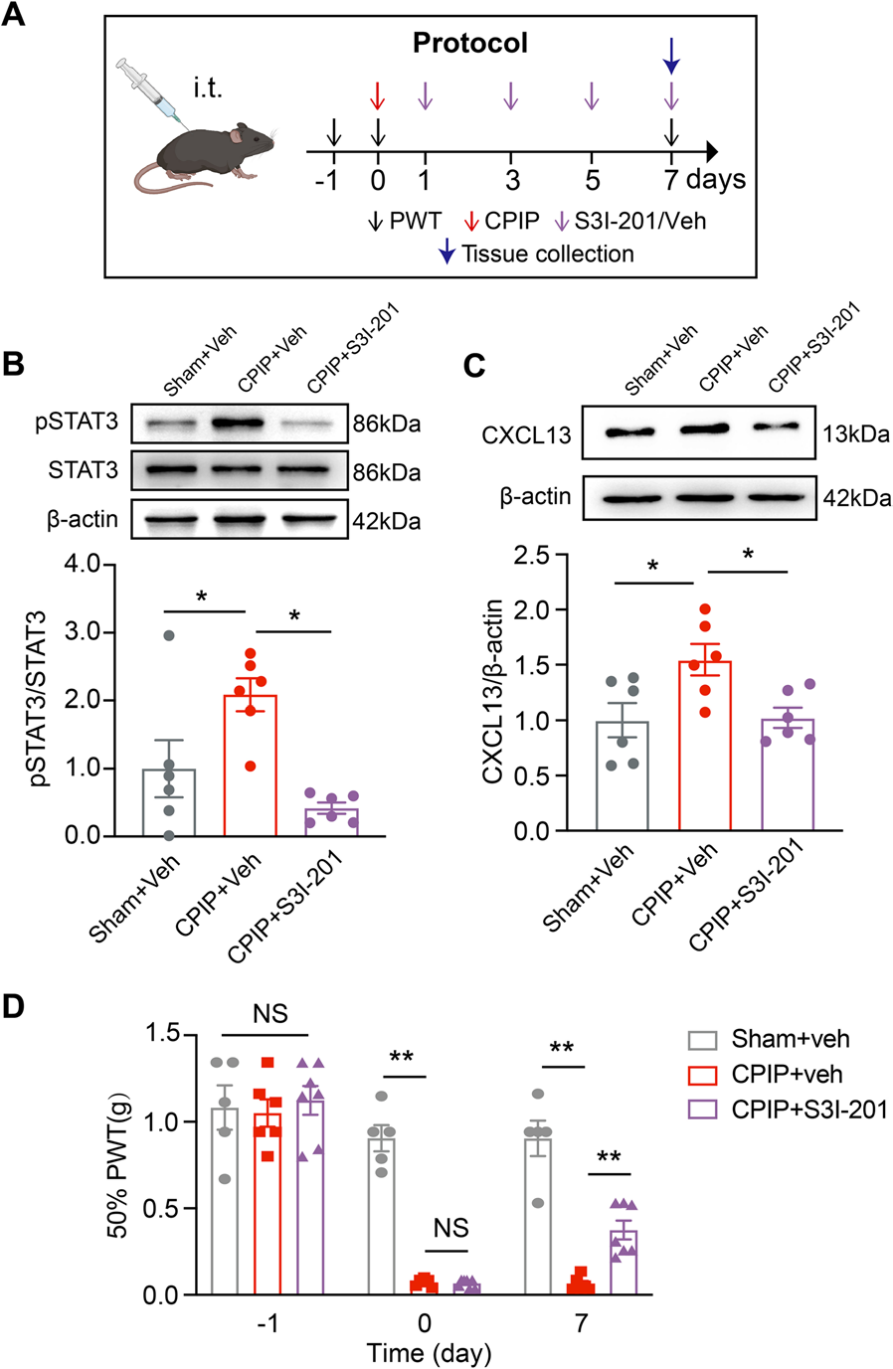
Activation of STAT3 is involved in CXCL13 upregulation in spinal cord dorsal horn and contributes to mechanical allodynia of CPIP mice. **A** Protocol illustrating specific time points for model establishment, intrathecal injections of NF-κB inhibitor PDTC/vehicle and behavioral test. **B** Western blot showing the expression of p-STAT3 and STAT3 in Sham + Veh, CPIP + Veh and CPIP + S3I-201 groups. Upper panels indicate representative blot images and lower panels indicate pooled data. **C** Western blot showing the expression of CXCL13 in all three groups. **D** 50% PWT changes in ipsilateral hindpaws after intrathecal treatment with S3I-201 or corresponding vehicle in CPIP and sham group of mice *n* = 5–6 mice/group. *p < 0.05, **p < 0.01. One-way ANOVA followed by Tukey’s post hoc test was used for comparisons in **B**, **C**. Two-way ANOVA with repeated measures followed by Tukey’s post hoc test was used for comparisons in **D**

We then examined the contribution of STAT3 activation to CXCL13 upregulation in SCDH. S3I-201, a specific STAT3 inhibitor, was applied intrathecally to CPIP model mice at time points indicated in Fig. 7A. S3I-201 treatment (10 μg/10 μl, i.t.) significantly reduced p-STAT3 upregulation in ipsilateral SCDH of CPIP mice, confirming the effectiveness of the drug we used (Fig. 7B). S3I-201 treatment reduced the overproduction of CXCL13 in ipsilateral SCDH of CPIP mice (Fig. 7C). S3I-201 significantly alleviated mechanical allodynia of CPIP mice vs. vehicle-treated group (Fig. 7D). These data indicate that STAT3 activation contributes to the upregulation of CXCL13 in SCDH and mechanical allodynia of CPIP mice.

### CXCR5-mediated NF-κB activation in spinal neurons contributes to mechanical allodynia of CPIP mice via inflammatory cytokine production

**Fig. 8:**
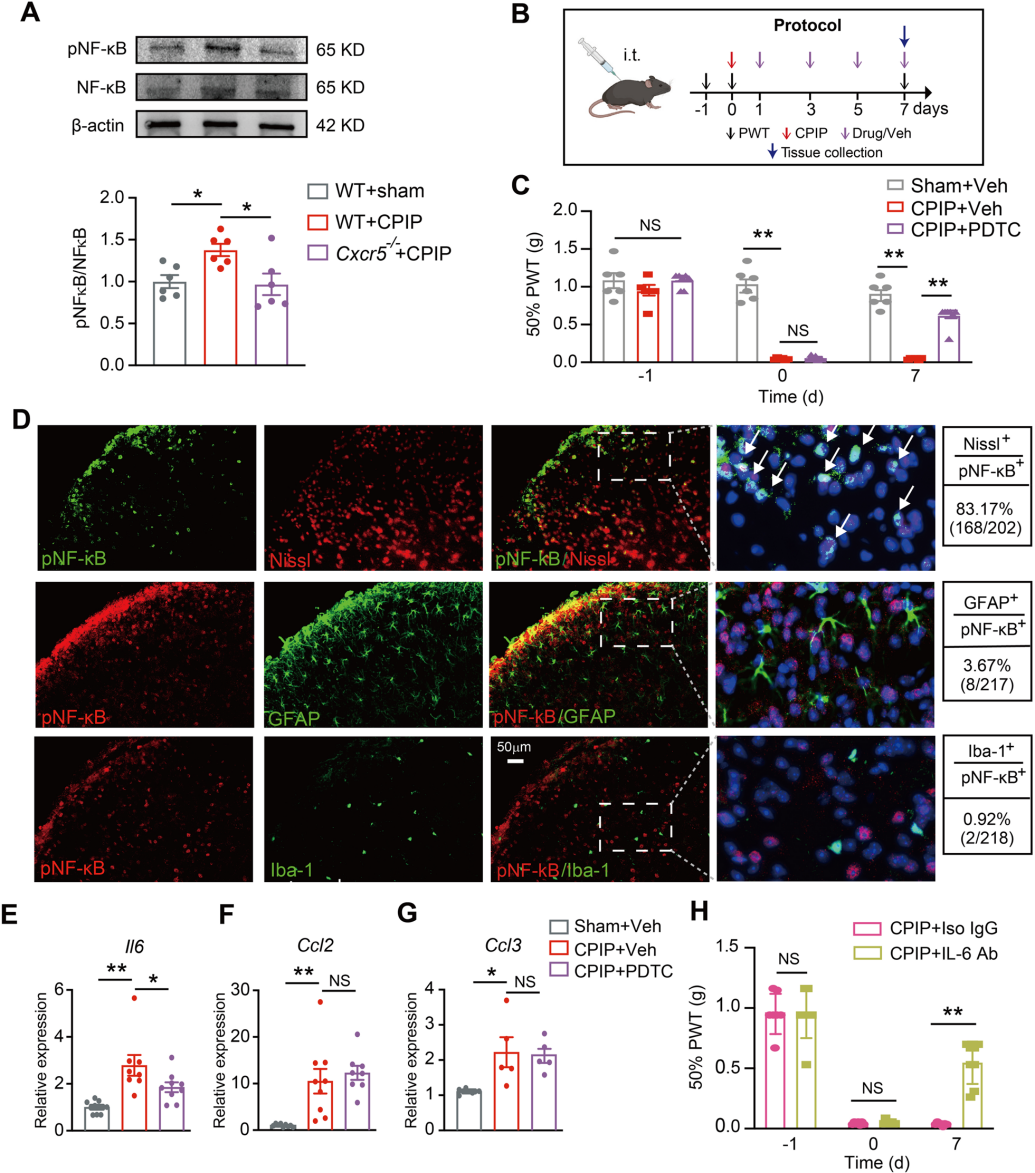
CXCR5-mediated NF-κB phosphorylation contributes to mechanical allodynia of CPIP mice via upregulating pro-inflammatory cytokine in SCDH. **A** Western blot showing expression of pNF-κB and NF-κB in ipsilateral SCDH of WT + sham, WT + CPIP and *Cxcr5*^−/−^ + CPIP groups. Upper panel indicate representative blot images and lower panel indicates pooled data. **B** Experimental protocol illustrating time points for behavioral test and intrathecal injection of NF-κB inhibitor PDTC (300 μg/5 μL)/vehicle (20% DMSO + 80% PBS) or IL-6 neutralizing antibody (10 ng/10 μl)/isotype control IgG (in PBS). **C** 50% PWT changes in ipsilateral hindpaw after intrathecal PDTC or vehicle in CPIP and sham group mice. **D** Double immunostaining of pNF-κB with Nissl, GFAP or Iba-1 in ipsilateral SCDH of CPIP mice. The dashed box area is further enlarged and shown on the right panel. White arrows denote co-localization. DAPI staining is shown in purple. Scale bar indicates 50 μm. **E**–**G** Gene expressions of *Il6*, *Ccl2* and *Ccl3* in ipsilateral SCDH of 3 groups by qPCR. **H** 50% PWT of ipsilateral hindpaws of CPIP mice treated with IL-6 neutralizing antibody or corresponding vehicle. *p < 0.05, **p < 0.01. *n* = 5–9 mice/group. Two-way ANOVA with repeated measures followed by Tukey’s post hoc test was used for comparisons in **C**, **H**. One-way ANOVA followed by Tukey’s post hoc test was used for comparisons in others

CXCL13 binds with CXCR5 to initiate several key intracellular signaling, including ERK, JNK and NF-κB pathway, etc. [16, 45, 46]. To further understand the downstream signaling of spinal CXCR5 in CPIP condition, we first checked ERK pathway. Western blot and immunostaining indicated phosphorylated ERK (p-ERK) expression was not significantly changed in ipsilateral SCDH of CPIP mice vs. sham mice (Additional file 9: Fig. S9A, B). In contrast, pNF-κB expression was significantly increased on Day 7 in ipsilateral SCDH of CPIP mice vs. sham mice (Fig. 8A). Moreover, *Cxcr5*^−/−^ mice showed significantly reduced pNF-κB expression vs. WT mice in CPIP condition (Fig. 8A). As a control, total NF-κB level remained unchanged (Fig. 8A). This result indicates that spinal pNF-κB is a downstream signaling of CXCR5 in the context of CPIP. We next examined whether NF-κB activation contributes to mechanical allodynia of CPIP mice. We injected NF-κB inhibitor pyrrolidine dithiocarbamate (PDTC) via intrathecal rout at time points as shown in Fig. 8B. PDTC treatment significantly reduced mechanical allodynia of CPIP model mice compared with vehicle treatment (Fig. 8C). We next explored the cellular distribution of pNF-κB in ipsilateral SCDH of CPIP model mice by triple fluorescence staining. We found that pNF-κB immunofluorescence mainly existed in DAPI positively labeled nuclear region that correlates well with anticipated pNF-κB location in cell nucleus (Fig. 8D). Immunostaining further showed that pNF-κB was exclusively co-expressed with Nissl-labeled neurons but barely with GFAP-labeled astrocytes or Iba1-labeled microglia, in ipsilateral SCDH of CPIP mice (Fig. 8D).

NF-κB activation induces expression of a variety of pro-inflammatory genes and thereby controls inflammation. We explored whether NF-κB might control pro-inflammatory gene expression in CPIP condition. We found that certain pro-inflammatory genes, including *Il6*, *Ccl2* and *Ccl3*, were significantly increased in ipsilateral SCDH of CPIP mice compared with sham mice (Fig. 8E–G). Among these genes, the expression of *Il6* was significantly reduced by PDTC treatment (Fig. 8E), indicating the expression of these two genes were regulated by NF-κB. We proceeded to test whether spinal blocking IL-6 could alleviate mechanical allodynia in CPIP mice. CPIP mice received intrathecal injection of monoclonal neutralizing antibodies for IL-6 or corresponding vehicle/isotype control IgG at time points indicated in Fig. 8B. Spinal blocking IL-6 significantly alleviated mechanical allodynia of CPIP mice compared with vehicle control at time points we observed (Fig. 8H). In together, these above results indicate that CXCL13/CXCR5 signaling contributes to mechanical allodynia of CPIP mice via activating NF-κB signaling in spinal neurons that contributes to pro-inflammatory cytokine production.

### Spinal CXCL13 application triggered CXCR5-dependent NF-κB activation and mechanical allodynia in naïve mice

**Fig. 9:**
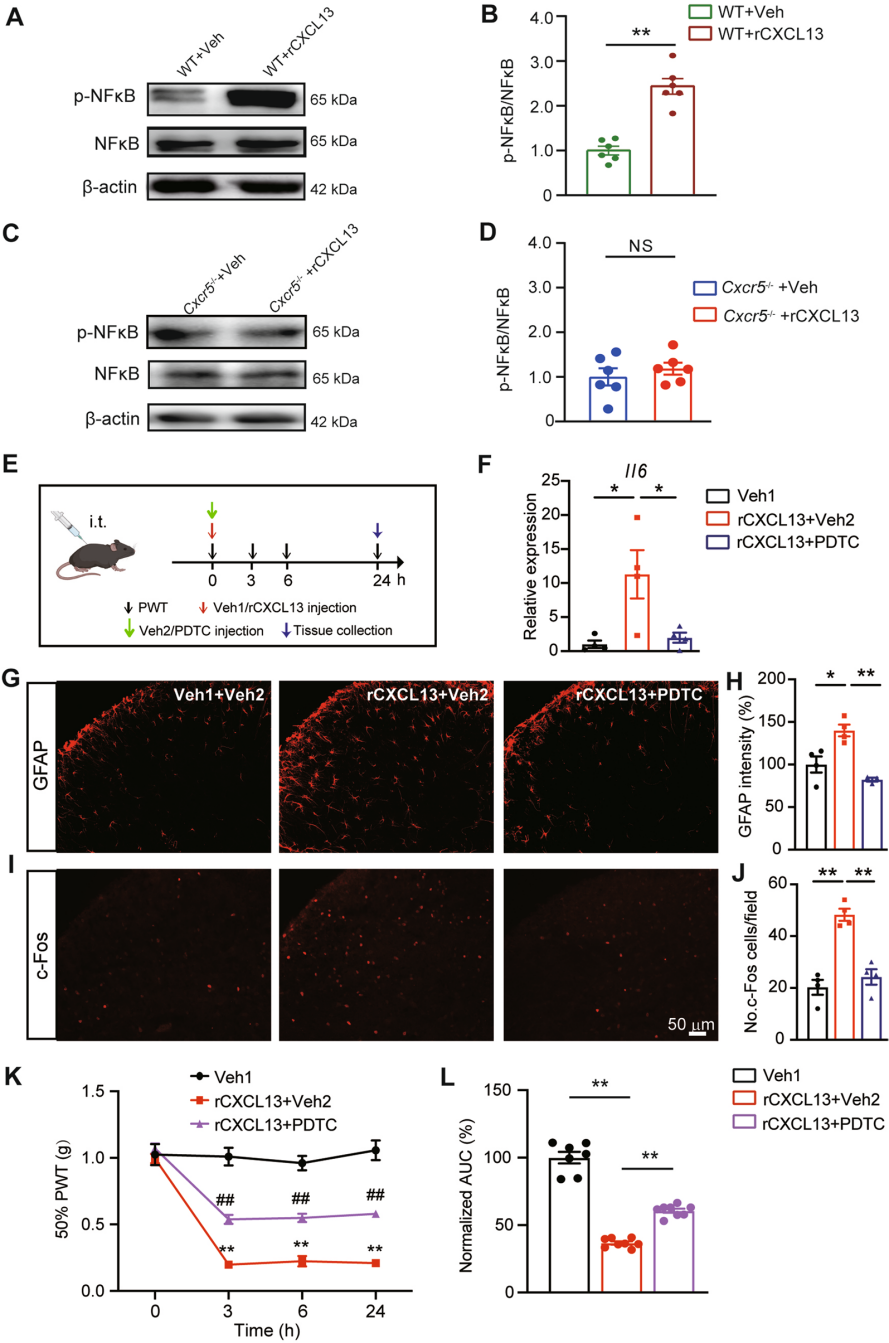
Intrathecal CXCL13 injection induces CXCR5-dependent NF-κB activation in spinal cord and results in mechanical allodynia in naïve mice. **A** Western blot gels showing pNF-κB and NF-κB expressions upon intrathecal injection of recombinant CXCL13 (rCXCL13, 100 ng/10 μL) or vehicle (1% BSA). **B** Summary of Western blot data in **A**. **C** Western blot gels showing expression of pNF-κB, NF-κB in spinal cord tissues from *Cxcr5*^−/−^ mice 24 h after CXCL13 or vehicle injection. **D** Summary of Western blot data in **C**. **p < 0.01. **E** Time points for intrathecal rCXCL13/Veh1 (1% BSA in PBS), PDTC/Veh2 (20% DMSO in PBS) injection, behavioral test and tissue collection in mice. **F** qPCR results showing *Il6* gene expression in SCDH of Veh1 + Veh2, rCXCL13 + Veh2 and rCXCL13 + PDTC group of mice. **G** Representative photos showing GFAP staining in SCDH of 3 groups of mice. **H** Summarized GFAP staining intensity/field in SCDH of 3 groups of mice. **I** Representative photos showing c-Fos staining in SCDH of 3 groups of mice. Scale bar indicates 50 μm. **J** Summarized No. of c-Fos stained cells/field in SCDH of 3 groups of mice. **K** Time courses showing 50% PWT changes in Veh1 + Veh2, rCXCL13 + Veh2 or rCXCL13 + PDTC groups of mice. **p < 0.01 vs. Veh1 group. ^##^*p* < 0.01 vs. rCXCL13 + Veh2 group. **L** Summary of normalized AUC of curves in **E**. *n* = 4–8 mice/group. Two-way ANOVA with repeated measures followed by Tukey’s post hoc test was used for comparison in **K**. One-way ANOVA followed by Tukey’s post hoc test was used for comparison in **F**, **H**, **J**, **L**. Student’s *t* test was used for comparisons in **B**, **D**

Intrathecal CXCL13 administration to naïve mice induces pain hypersensitivity via CXCR5 [16]. We then asked whether intrathecal CXCL13 administration to naïve mice would result in NF-κB activation in spinal cord via CXCR5-dependent signaling. We found that intrathecal CXCL13 injection produced significant pNF-κB upregulation in spinal cord of naïve mice vs. vehicle-injected mice (Fig. 9A, B). In contrast, intrathecal CXCL13 injection into *Cxcr5*^−/−^ mice did not result in any obvious pNF-κB upregulation (Fig. 9C, D). Intrathecal CXCL13 injection triggered significant gene expression upregulation of pro-inflammatory cytokine *Il6* in SCDH of naïve mice, whereas this upregulation was significantly attenuated by blocking NF-κB signaling using PDTC (Fig. 9E, F). We further monitored the astrocyte and neuronal activation in SCDH by immunostaining. We found that intrathecal CXCL13 triggered c-Fos and GFAP activation in SCDH, which was reduced by PDTC treatment (Fig. 9G–J). We continued to test whether blocking NF-κB activation would attenuate CXCL13-induced mechanical allodynia in naïve mice. We found that co-treatment of NF-κB inhibitor PDTC with CXCL13 significantly abolished CXCL13-induced mechanical allodynia in naïve mice (Fig. 9K, L). Therefore, these results indicate that intrathecal CXCL13 application can activate CXCR5 and downstream NF-κB signaling in spinal cord of naïve animal to produce mechanical allodynia in naïve mice.

### Specific overexpression of CXCL13 in SCDH neurons is sufficient to induce persistent mechanical allodynia in naïve mice

**Fig. 10:**
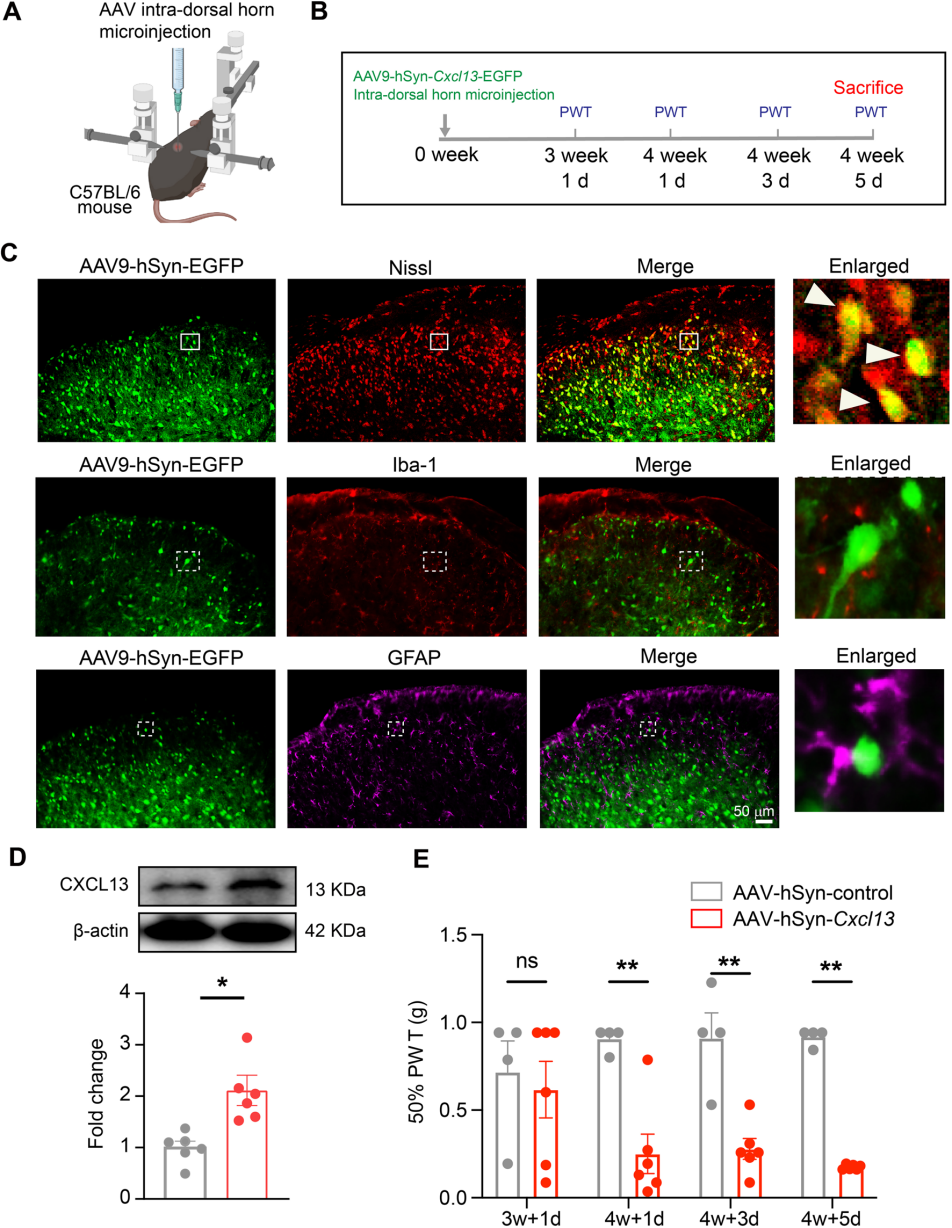
Specific overexpression of CXCL13 in SCDH neurons induces mechanical allodynia in naïve mice. **A** Cartoon showing intra-dorsal horn microinjection of AAV9-hSyn-*Cxcl13*-EGFP or control AAV in naïve C57BL/6 mice. **B** Time schedule of the experiments. **C** Fluorescence images showing EGFP signal co-localized with Nissl (indicated by white arrowheads), but not with GFAP or Iba-1 in SCDH of AAV-injected mice. **D** Western blot showing an increased CXCL13 expression in SCDH from AAV-hSyn-*Cxcl13*-EGFP injected mice compared with control AAV-injected mice. **E** Specific overexpression of CXCL13 in SCDH neurons induced mechanical allodynia in mice. *n* = 4–6 mice/group. *p < 0.05, **p < 0.01. Student’s *t* test was used for comparisons in **D**. Two-way ANOVA with repeated measures followed by Tukey’s post hoc test was used for comparison in **E**

To address whether neuron-derived CXCL13 could contribute to mechanical allodynia, adeno-associated virus 9 (AAV9) expressing *Cxcl13* gene driven by neuronal promoter hSyn was intra-dorsal horn microinjected to specifically overexpress CXCL13 in SCDH neurons (Fig. 10A, B). Intra-dorsal horn microinjection approach enables localized viral infection only in SCDH region but not in DRG or other higher brain regions. As can be seen in Fig. 10C, 5 weeks after intra-dorsal horn microinjection, AAV9-hSyn-EGFP showed obvious expression only in Nissl-labeled SCDH neurons, but not in GFAP- or Iba-1-labeled glial cells, demonstrating the specificity of AAV infection. Furthermore, CXCL13 protein level was significantly increased in AAV-hSyn-*Cxcl13*-injected group compared with control AAV-injected group (Fig. 10D; Additional file 11). More importantly, AAV-hSyn-*Cxcl13*-injected group showed obvious mechanical allodynia starting from 4 weeks after viral infection (Fig. 10E). These results suggest that CXCL13 derived from SCDH neurons is sufficient to induce persistent mechanical allodynia in naïve mice.

## Discussion

Neuroinflammation plays a crucial role in mediating chronic pain [10]. Growing evidence demonstrates chemokines are important participants in the process of neuroinflammation [12, 13]. Recent studies showed CXCL13 is among one of the chemokines that contributes to the pathogenesis of several chronic pain conditions via mediating spinal neuroinflammation [16, 19]. Here, we observed that expression of CXCL13 was significantly increased in ipsilateral SCDH of CPIP mice. Behavioral experiments further showed that blocking CXCL13 by neutralizing antibody effectively reduced mechanical allodynia of CPIP mice, indicating a critical role of spinal CXCL13 in pain mechanism of CPIP. However, the mechanisms underlying how CXCL13 expression is modulated under chronic pain condition still remain largely unknown. STAT3 serves as an important transcription factor to modulate chemokine or cytokine expression in nerve system and contributes to chronic pain [47–50]. We found that p-STAT3 was persistently increased in ipsilateral SCDH of CPIP mice. Pharmacological blocking STAT3 reduced spinal CXCL13 overexpression as well as mechanical allodynia. p-STAT3 also co-expressed with CXCL13 in SCDH neurons. These results demonstrate STAT3 contributes to CXCL13 overproduction in spinal neurons during CPIP.

*Cxcr5*^−/−^ mice showed remarkable deficits in mechanical and cold allodynia as well as affective response vs. WT mice in CPIP condition. Several signaling pathways have been documented to connect with CXCR5, including ERK, NF-κB, etc. [45]. ERK is a downstream signaling of CXCL13/CXCR5 in a mouse model of neuropathic pain [16]. But we found ERK was not activated in ipsilateral SCDH of CPIP model mice, thus ruling out possible involvement of ERK in CXCR5 signaling under CPIP condition. Instead, we found that pNF-κB expression was significantly increased in ipsilateral SCDH of CPIP mice. We further showed that in *Cxcr5*^−/−^ mice, NF-κB activation was reduced in ipsilateral SCDH in CPIP condition. NF-κB inhibitor PDTC attenuated mechanical allodynia of CPIP mice. We found p-NF-κB was exclusively expressed in spinal neurons but not in astrocytes or microglia. Therefore, these results indicate that neuronal NF-κB is a possible downstream signal of CXCL13/CXCR5 in CPIP model animals.

NF-κB is a key transcription factor that controls expression of a variety of pro-inflammatory genes and has important roles in neuroinflammation and chronic pain [51]. NF-κB activation in spinal neurons may trigger the production of inflammatory cytokines that contributes to chronic pain in CPIP model mice. We found that the gene expression of pro-inflammatory cytokine *Il6* was significantly increased in ipsilateral SCDH of CPIP mice, which was controlled by NF-κB signaling. It is known that IL-6 can be produced from spinal neurons upon noxious stimulation and contributes to chronic pain via mediating neuron–glial cell crosstalk and neuroinflammation [52–54]. We found that spinal blocking IL-6 significantly alleviated mechanical allodynia of CPIP mice. We further showed that NF-κB was activated in spinal cord by intrathecal CXCL13 injection into naïve animals via CXCR5-dependent manner. Blocking NF-κB attenuates CXCL13-induced *Il6* gene upregulation as well as mechanical allodynia in naïve mice. These results suggest CXCL13/CXCR5 contributes to mechanical allodynia of CPIP mice by activating NF-κB-mediated pro-inflammatory cytokine production in SCDH.

Given the fact that p-STAT3, CXCL13/CXCR5 and the downstream signaling pNF-κB was exclusively expressed in spinal neurons of CPIP model mice, we propose that CXCL13 is upregulated via pSTAT3-dependent mechanism in SCDH neurons in CPIP condition. Neuronal-derived CXCL13 further acts on CXCR5 that is predominantly expressed in spinal neurons via autocrine manner to initiate downstream NF-κB signaling. NF-κB activation in spinal neurons produces pro-inflammatory cytokine IL-6, which may further activate spinal glial cells to initiate neuronal–glial crosstalk and mediate chronic pain in CPIP condition.

In this work, we found CXCR5 was predominantly expressed in spinal neurons, which was consistent with one recent study [55], however, we also observed CXCR5 expression in spinal astrocytes, although to a much less extent. We found pNF-κB was not expressed in spinal astrocytes, ruling out involvement of astrocytic pNF-κB signaling in contributing to chronic pain of CPIP model mice. But it still remains possible that some other signaling may be activated in astrocytes upon CXCR5 activation and contributes to pain mechanism of CPIP model mice. Therefore, future studies that can specifically target CXCR5 expression in neurons or astrocytes will be needed to fully address the cellular mechanisms underlying chronic pain mechanisms of CPIP model mice.

Sex dimorphism has become increasingly recognized in pain field. Epidemiology data indicate that female population has a higher incidence of CRPS-I than male [56]. Emerging evidence indicates that sex difference stems from the process of neuron-immune regulation of pain in which the contribution of microglia vs. astrocytes to chronic pain differs [57]. Spinal microglia selectively initiate sex-dependent pain signaling in male animals of chronic pain models [58–60], whereas spinal astrocytes seems to be independent of sex [61]. However, it should be noted that sex-dependent microglial signaling may not happen in all pain conditions. For instance, it is reported that spinal microglia contribute to bone cancer pain in female rats [62]. In our study, we found no difference between female and male mice in CPIP-induced mechanical allodynia, a result consistent with a prior study [37]. Furthermore, we found that female *Cxcr5*^−/−^ mice showed similar deficits in mechanical allodynia as well as reductions in spinal glial cell overactivation and neuronal activation compared with male mice in CPIP condition. This result demonstrates that CXCL13/CXCR5 signaling mediates mechanical allodynia in CPIP mice via sex-independent mechanism.

CPIP mice displayed contralateral mechanical allodynia, a sign of mirror-image pain (MIP). This observation is consistent with CRPS-I patients who also showed MIP [63]. Currently, the mystery underlying MIP is far from being understood. Peripheral and spinal mechanisms are both proposed to contribute to MIP [39, 64]. Blocking spinal glia activation and pro-inflammatory cytokines, including TNF-α, IL-1β, and IL-6, significantly alleviate MIP of neuropathic pain model animals. We found that CXCL13/CXCR5 expression was not significantly upregulated in contralateral SCDH of CPIP group vs. sham group, excluding direct participation of contralateral CXCL13/CXCR5 signaling in MIP. We further found that CXCL13 acts upon CXCR5 to trigger *Il6* gene upregulation in ipsilateral SCDH of CPIP mice via NF-κB-dependent mechanism. Thus, although CXCL13/CXCR5 signaling was not activated in contralateral side, it remains likely that IL-6 and/or other inflammatory mediators produced from ipsilateral side may diffuse to contralateral side via cerebrospinal fluid and activate spinal glia cells and contribute to MIP of CPIP mice. This hypothesis may explain why *Cxcr5*^−/−^ mice showed reduced mechanical allodynia in contralateral side as well as reduced glial cell activation and c-Fos^+^ cell number in contralateral SCDH in CPIP condition. Further efforts will be needed to test this hypothesis.

## Conclusions

This study demonstrates a previous unidentified role of CXCL13/CXCR5 signaling in mediating spinal neuroinflammation and mechanical pain in an animal model of CRPS-I. Our work suggests that targeting CXCL13/CXCR5 pathway may lead to novel therapeutic approaches for treating CRPS-I.

## Supplementary Information

**Additional file 1: Figure S1.** The establishment of the mouse model of CRPS-I.Representative pictures showing the hind paw in different time points after model establishment. The red arrow indicates O-ring location.Percent increase in paw thickness of ipsilateral sideand contralateral side.50% paw withdrawal thresholdmeasured in contralateral hindpaw before and after model establishment.Normalized AUC analysis of curves shown in panel. n = 6 mice/group. *p < 0.05, **p < 0.01 vs. sham group. NS: no significance. Two-way ANOVA with repeated measures followed by Tukey’s post hoc test was used for comparisons in panels B, C, D. Student’s t test was used for comparisons in panels E.

**Additional file 2: Figure S2.** CXCL13 and CXCR5 expression in contralateral SCDH of CPIP model mice.Western blot examination of CXCL13and CXCR5in contralateral SCDH of sham and CPIP model mice on day 0, 7 14.Immunostaining of CXCL13and CXCR5in contralateral SCDH of sham and CPIP model mice on day 14. NS: no significance. n = 4–5 mice/group. One-way ANOVA followed by Tukey’s post hoc test was used for comparisons in panels A&B. Student’s t test was used for comparisons in panels C&D.

**Additional file 3: Figure S3.** Validation of the CXCR5 antibody.Immunostaining of SCDH from wildtypemouse using CXCR5 antibody.Immunostaining of SCDH from Cxcr5−/− mouse using CXCR5 antibody. Enlarged pictures were shown on the right panels. Scale bar indicates 50 μm.

**Additional file 4: Figure S4.** Expressions of CXCL13 and CXCR5 were not significantly changed in ipsilateral dorsal root ganglion of CPIP mice.Western blot showing the expression of CXCL13and CXCR5in ipsilateral DRG of sham and CPIP mice on day 7 and 14 after model establishment. Upper panel indicates representative blot images and lower panel indicates pooled data.Immunostaining of CXCL13 in ipsilateral DRG of sham and CPIP mice on day 14 after CPIP model establishment.Summary of normalized results of CXCL13 immunostaining intensity per observation field. The result of sham group was normalized to 100%.Immunostaining of CXCR5 in ipsilateral DRG of sham and CPIP mice on day 14 after CPIP model establishment.Summarized normalized results of CXCR5 immunostaining intensity per observation field. NS: no significance. n = 5–6 mice/group. One-way ANOVA followed by Tukey’s post hoc test was used for comparisons in A&B. Student’s t test was used for comparisons in panels D&F.

**Additional file 5: Figure S5.** Typical signs of astrocytes and microglia upon activation in ipsilateral SCDH of CPIP model mice.Representative pictures showing morphological changes of astrocytesand microgliain ipsilateral SCDH of CPIP model mice upon activation compared with sham group mice.

**Additional file 6: Figure S6.** Cxcr5−/− mice showed normal locomotor activities in the open field test.Representative movement traces of WT and Cxcr5−/− mice in the open field test.Summary of travelled distances in center zone.Summary of time spent in center zone. Student’s t test was used for comparisons. n = 6 mice/group.

**Additional file 7: Figure S7.** Cxcr5−/− mice showed significantly attenuated mechanical allodynia in contralateral hind paw and reduced c-Fos and glial cell activation in contralateral SCDH in CPIP condition.Time course showing 50% PWT changes in contralateral hindpaws of WT and Cxcr5−/− mice after CPIP model establishment.Summary of normalized AUC of the curves in panel A. n = 6 mice/group.Immunostaining of GFAP, Iba-1and c-Fosin contralateral SCDH from WT + sham, WT + CPIP, Cxcr5−/− + sham and Cxcr5−/− + CPIP groups.Summary of GFAP& Iba-1fluorescence intensity and c-Fospositively stained cell number. n = 4–5 mice/group. *p < 0.05, **p < 0.01 vs. WT + sham group; #p < 0.05, ##p < 0.01 vs. WT + CPIP group. Scale bar indicates 50 μm. Two-way ANOVA with repeated measures followed by Tukey’s post hoc test was used for comparisons in panels A. One-way ANOVA followed by Tukey’s post hoc test was used for comparisons in panels B, F, G&H.

**Additional file 8: Figure S8.** CXCR5 is important for mechanical allodynia as well as c-Fos and glial cell overactivation in ipsilateral spinal cord dorsal horn of female CPIP model mice.Time course showing 50% PWT changes in ipsilateral hindpaws of WT and Cxcr5−/− female mice after model establishment.Summary of normalized AUC of curves in panel A.Time course showing 50% PWT changes in contralateral hindpaws of WT and Cxcr5−/− female mice after model establishment.Summary of normalized AUC of curves in panel C.Immunostaining of GFAP, Iba1and c-Fosin ipsilateral SCDH from WT + sham, WT + CPIP, Cxcr5−/− + sham and Cxcr5−/− + CPIP groups of female mice.Summary of GFAP& Iba1fluorescence intensity and c-Fospositively stained cell number. n = 5–6 mice/group. **p < 0.01 vs. WT + sham group; #p < 0.05, ##p < 0.01 vs. WT + CPIP group. Scale bar indicates 50 μm. Two-way ANOVA with repeated measures followed by Tukey’s post hoc test was used for comparisons in panels A&C. One-way ANOVA followed by Tukey’s post hoc test was used for comparisons in others.

**Additional file 9: Figure S9.** ERK was not activated in ipsilateral SCDH of CPIP mice.Western blot showing the expression of p-ERK in ipsilateral SCDH of sham and CPIP mice. Upper panels indicated the representative blot images, and lower panel indicate the pooled data.Immunostaining of p-ERK in ipsilateral SCDH of sham and CPIP mice on day 7. n = 5 mice/group. One-way ANOVA followed by Tukey’s post hoc test was used in panel A. Student’s t test was used in panel B.

**Additional file 10: Table S1.** Sequences of primers used for qPCR.

**Additional file 11: File 1.** Uncropped Western bot gel images.

## Data Availability

The key data are contained in the figures, tables, and additional files. The datasets used and/or analyzed during this study can be obtained from the corresponding author Dr. Boyi Liu on reasonable requests.

## Abbreviations

CPIP: Chronic post-ischemic pain
CRPS-I: Complex regional pain syndrome type-I
RNA-Seq: RNA-sequencing
PDTC: Ammonium pyrrolidinedithiocarbamate
SCDH: Spinal cord dorsal horn
DRG: Dorsal root ganglia
I.t.: Intrathecal
I.p.: Intraperitoneal
PWT: Paw withdrawal threshold
Ab: Antibody
NS: No significance
IR: Immunoreactivity
PEA: Real-time place escape/avoidance
AAV: Adeno-associated virus
