# Supplementary figures and images for: CXCL13 contributes to chronic pain of a mouse model of CRPS-I via CXCR5-mediated NF-κB activation and pro-inflammatory cytokine production in spinal cord dorsal horn
Source: J Neuroinflammation. 2023 May 8;20:109. doi: 10.1186/s12974-023-02778-x (PMC10165831; doi:10.1186/s12974-023-02778-x)

**Suppl. Fig.1**

**A**

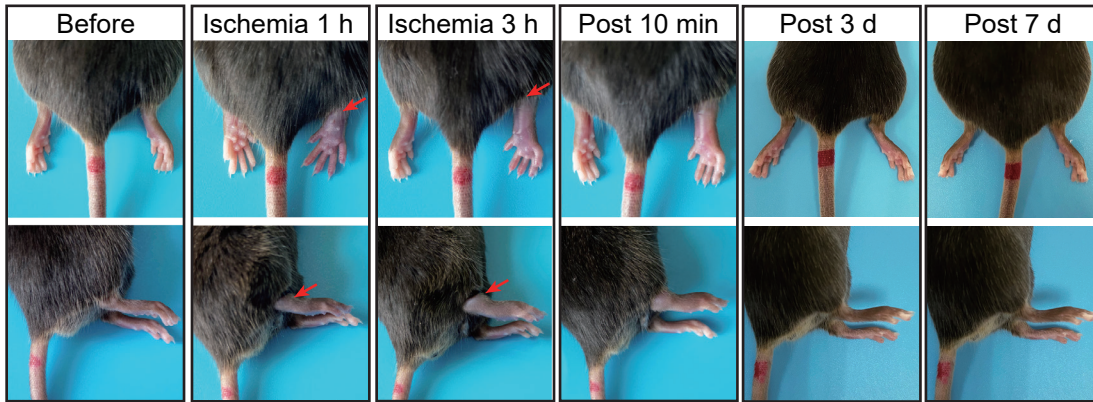

**B**

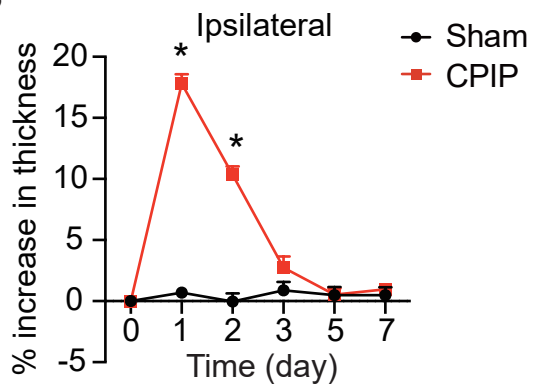

**C**

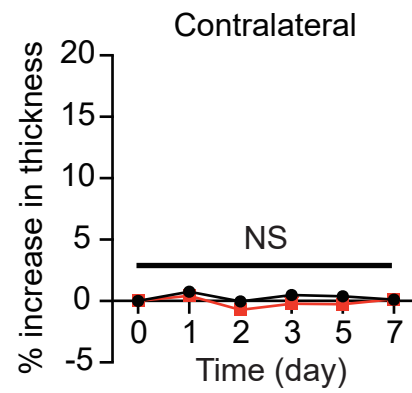

**D**

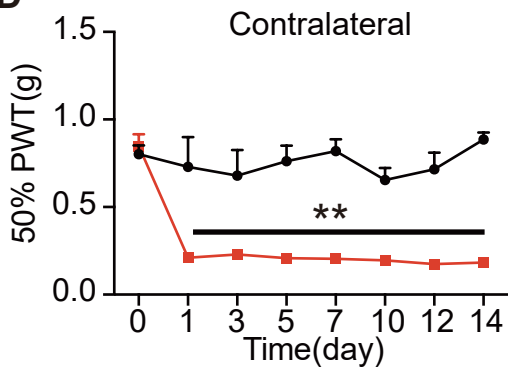

**E**

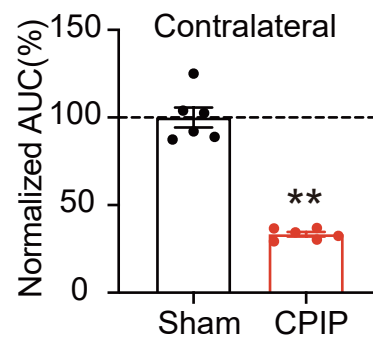

Supplement: Supplementary file 1 — Additional file 1: Figure S1. The establishment of the mouse model of CRPS-I.Representative pictures showing the hind paw in different time points after model establishment. The red arrow indicates O-ring location.Percent increase in paw thickness of ipsilateral sideand contralateral side.50% paw withdrawal thresholdmeasured in contralateral hindpaw before and after model establishment.Normalized AUC analysis of curves shown in panel. n = 6 mice/group. *p < 0.05, **p < 0.01 vs. sham group. NS: no significance. Two-way ANOVA with repeated measures followed by Tukey’s post hoc test was used for comparisons in panels B, C, D. Student’s t test was used for comparisons in panels E. [file 12974_2023_2778_MOESM1_ESM.pdf]

Suppl. Fig. 2

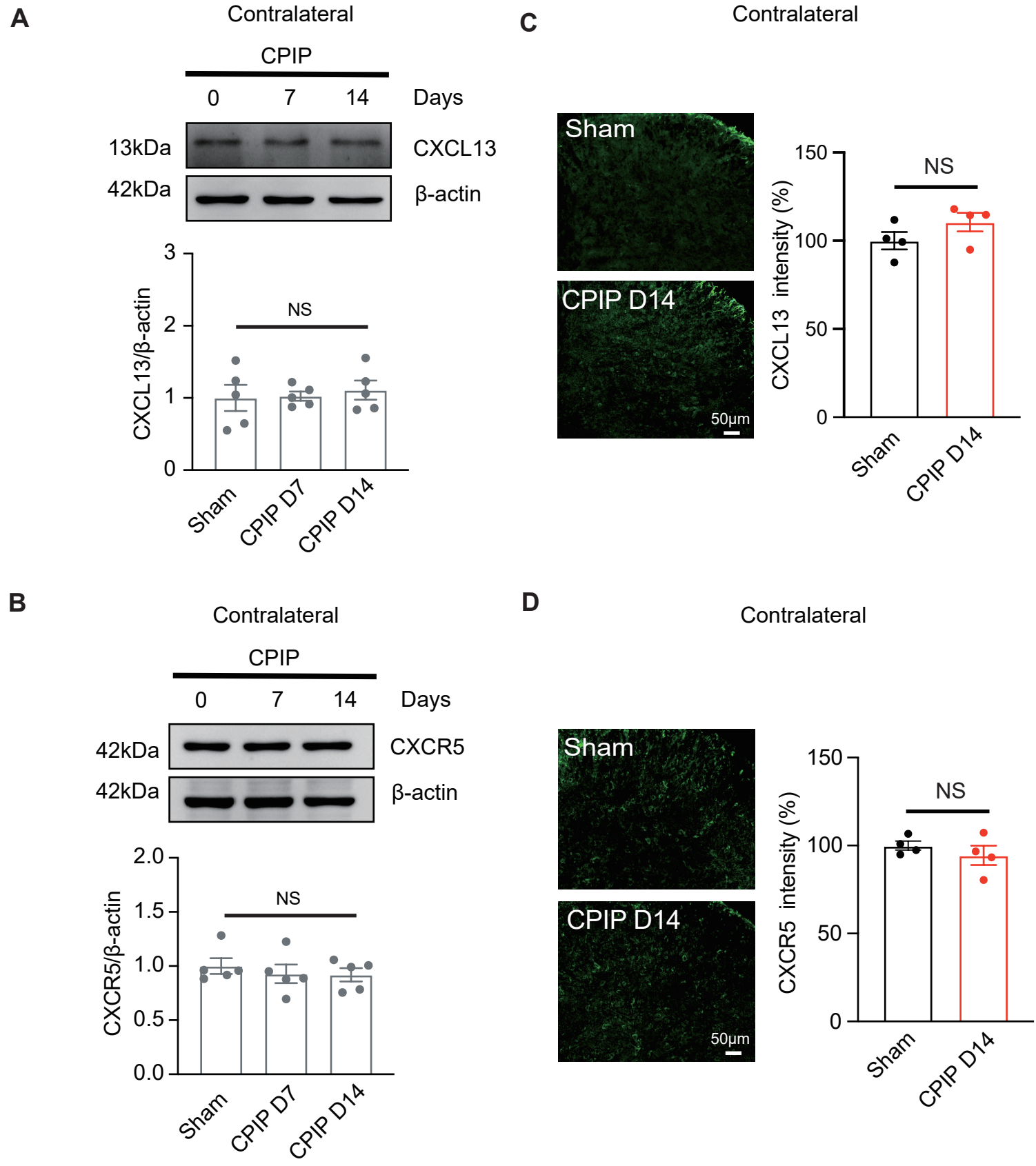

Supplement: Supplementary file 2 — Additional file 2: Figure S2. CXCL13 and CXCR5 expression in contralateral SCDH of CPIP model mice.Western blot examination of CXCL13and CXCR5in contralateral SCDH of sham and CPIP model mice on day 0, 7 14.Immunostaining of CXCL13and CXCR5in contralateral SCDH of sham and CPIP model mice on day 14. NS: no significance. n = 4–5 mice/group. One-way ANOVA followed by Tukey’s post hoc test was used for comparisons in panels A&B. Student’s t test was used for comparisons in panels C&D. [file 12974_2023_2778_MOESM2_ESM.pdf]

Suppl. Fig. 3

A

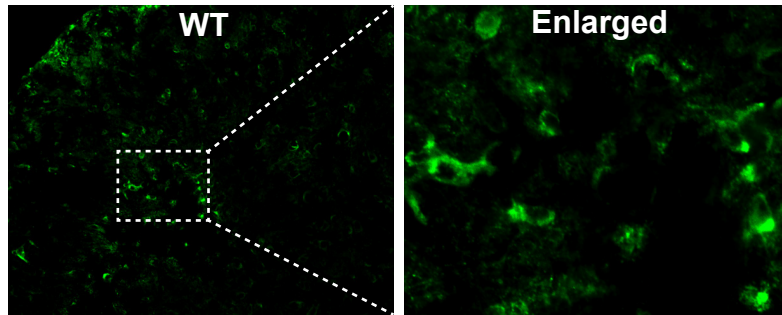

B

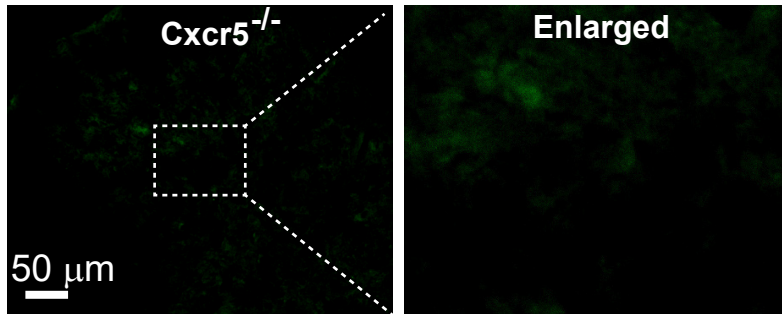

Supplement: Supplementary file 3 — Additional file 3: Figure S3. Validation of the CXCR5 antibody.Immunostaining of SCDH from wildtypemouse using CXCR5 antibody.Immunostaining of SCDH from Cxcr5−/− mouse using CXCR5 antibody. Enlarged pictures were shown on the right panels. Scale bar indicates 50 μm. [file 12974_2023_2778_MOESM3_ESM.pdf]

**Suppl. Fig. 4**

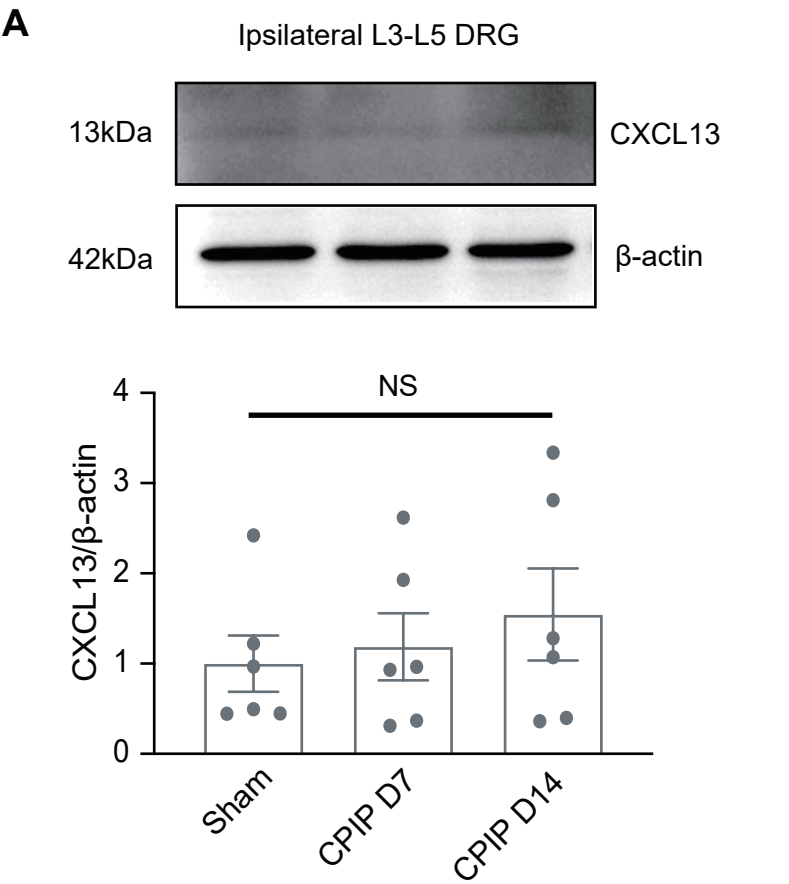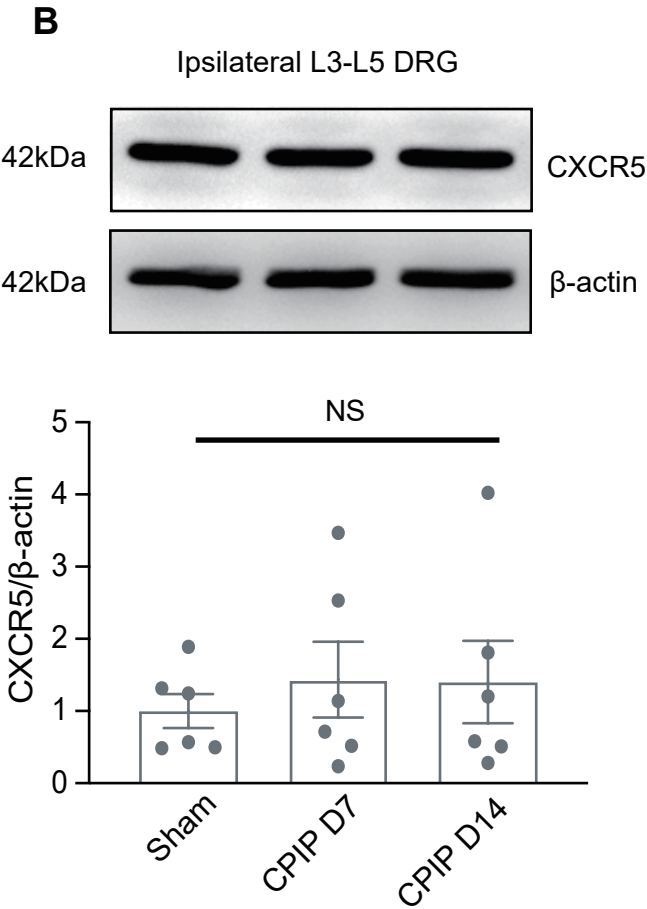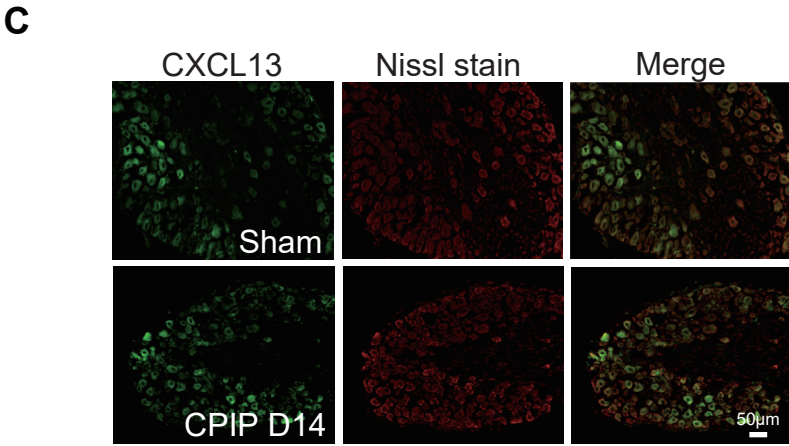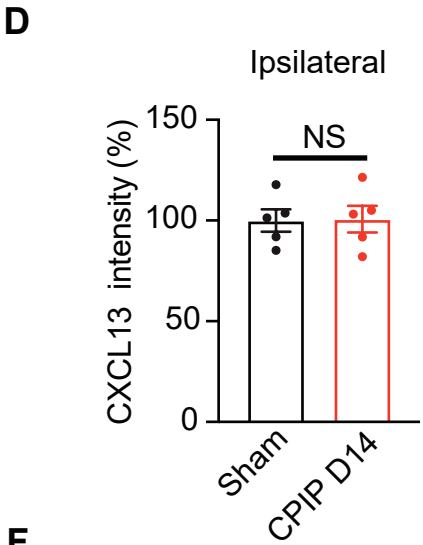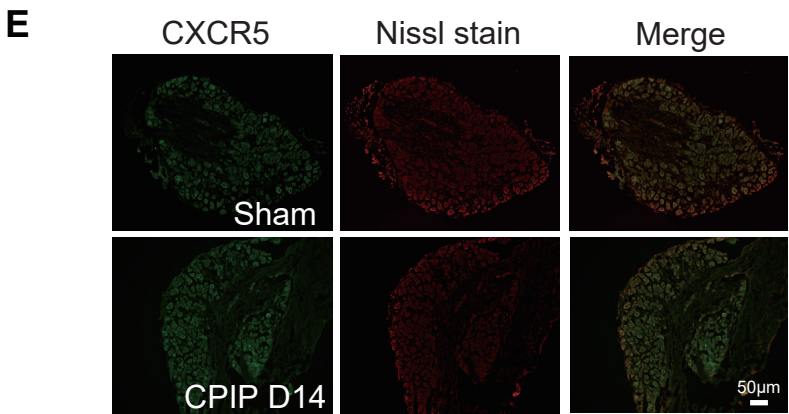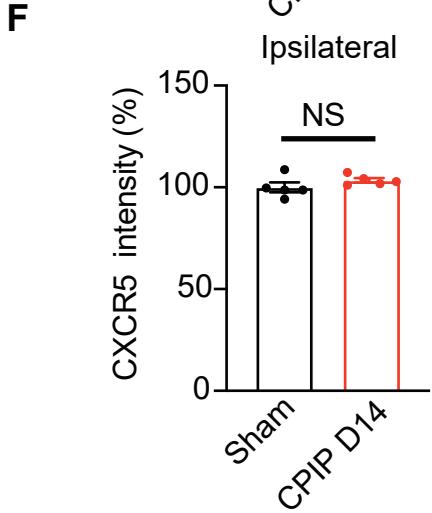

Supplement: Supplementary file 4 — Additional file 4: Figure S4. Expressions of CXCL13 and CXCR5 were not significantly changed in ipsilateral dorsal root ganglion of CPIP mice.Western blot showing the expression of CXCL13and CXCR5in ipsilateral DRG of sham and CPIP mice on day 7 and 14 after model establishment. Upper panel indicates representative blot images and lower panel indicates pooled data.Immunostaining of CXCL13 in ipsilateral DRG of sham and CPIP mice on day 14 after CPIP model establishment.Summary of normalized results of CXCL13 immunostaining intensity per observation field. The result of sham group was normalized to 100%.Immunostaining of CXCR5 in ipsilateral DRG of sham and CPIP mice on day 14 after CPIP model establishment.Summarized normalized results of CXCR5 immunostaining intensity per observation field. NS: no significance. n = 5–6 mice/group. One-way ANOVA followed by Tukey’s post hoc test was used for comparisons in A&B. Student’s t test was used for comparisons in panels D&F. [file 12974_2023_2778_MOESM4_ESM.pdf]

## Suppl. Fig. 5

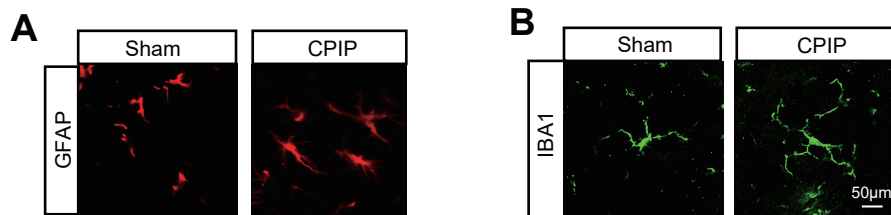

Supplement: Supplementary file 5 — Additional file 5: Figure S5. Typical signs of astrocytes and microglia upon activation in ipsilateral SCDH of CPIP model mice.Representative pictures showing morphological changes of astrocytesand microgliain ipsilateral SCDH of CPIP model mice upon activation compared with sham group mice. [file 12974_2023_2778_MOESM5_ESM.pdf]

Suppl. Fig. 6

**A**

WT

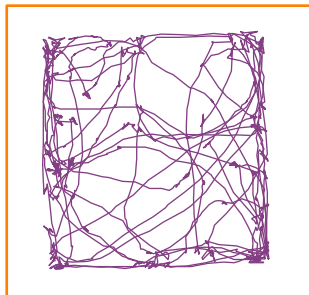

**B**

*Cxcr5*<sup>-/-</sup>

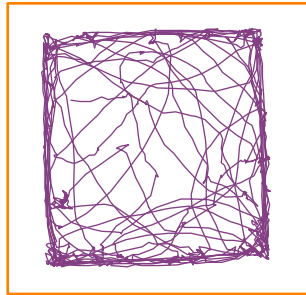

**C**

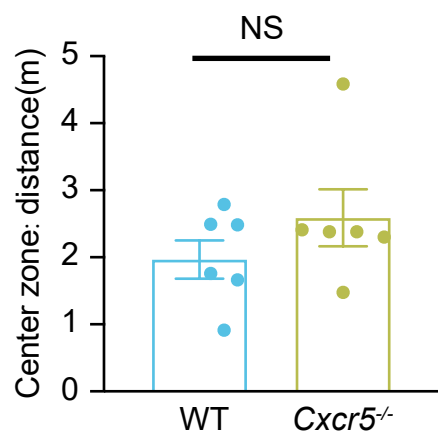

**D**

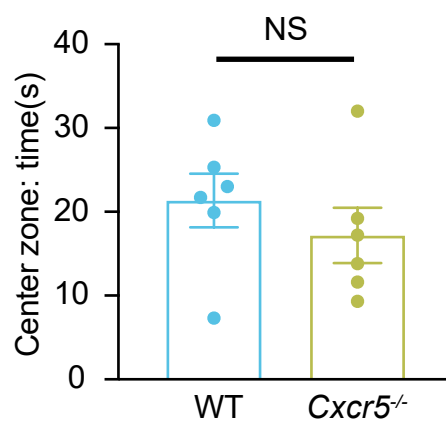

Supplement: Supplementary file 6 — Additional file 6: Figure S6. Cxcr5−/− mice showed normal locomotor activities in the open field test.Representative movement traces of WT and Cxcr5−/− mice in the open field test.Summary of travelled distances in center zone.Summary of time spent in center zone. Student’s t test was used for comparisons. n = 6 mice/group. [file 12974_2023_2778_MOESM6_ESM.pdf]

## Suppl. Fig. 7

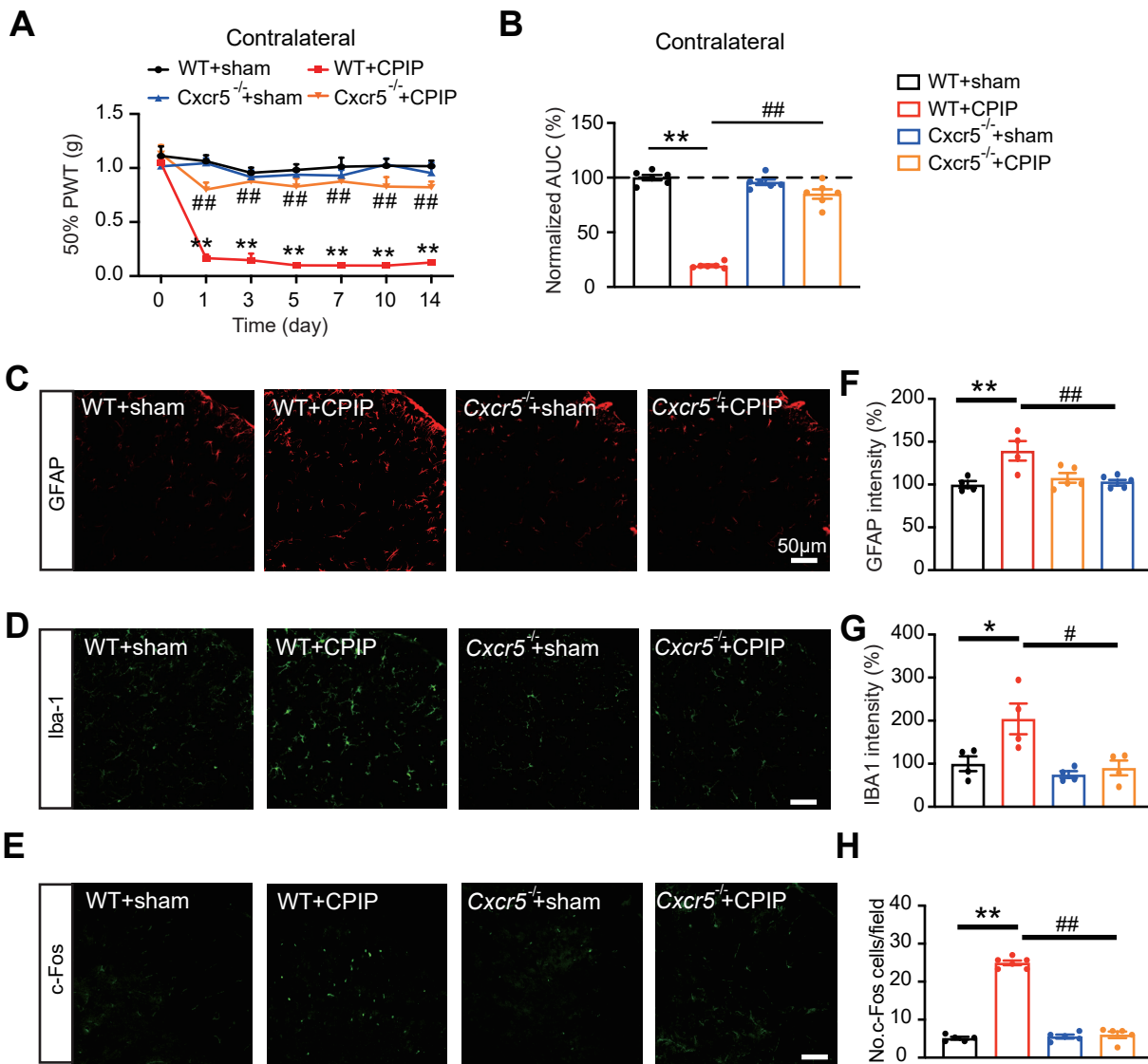

Supplement: Supplementary file 7 — Additional file 7: Figure S7. Cxcr5−/− mice showed significantly attenuated mechanical allodynia in contralateral hind paw and reduced c-Fos and glial cell activation in contralateral SCDH in CPIP condition.Time course showing 50% PWT changes in contralateral hindpaws of WT and Cxcr5−/− mice after CPIP model establishment.Summary of normalized AUC of the curves in panel A. n = 6 mice/group.Immunostaining of GFAP, Iba-1and c-Fosin contralateral SCDH from WT + sham, WT + CPIP, Cxcr5−/− + sham and Cxcr5−/− + CPIP groups.Summary of GFAP& Iba-1fluorescence intensity and c-Fospositively stained cell number. n = 4–5 mice/group. *p < 0.05, **p < 0.01 vs. WT + sham group; #p < 0.05, ##p < 0.01 vs. WT + CPIP group. Scale bar indicates 50 μm. Two-way ANOVA with repeated measures followed by Tukey’s post hoc test was used for comparisons in panels A. One-way ANOVA followed by Tukey’s post hoc test was used for comparisons in panels B, F, G&H. [file 12974_2023_2778_MOESM7_ESM.pdf]

Suppl. Fig. 8

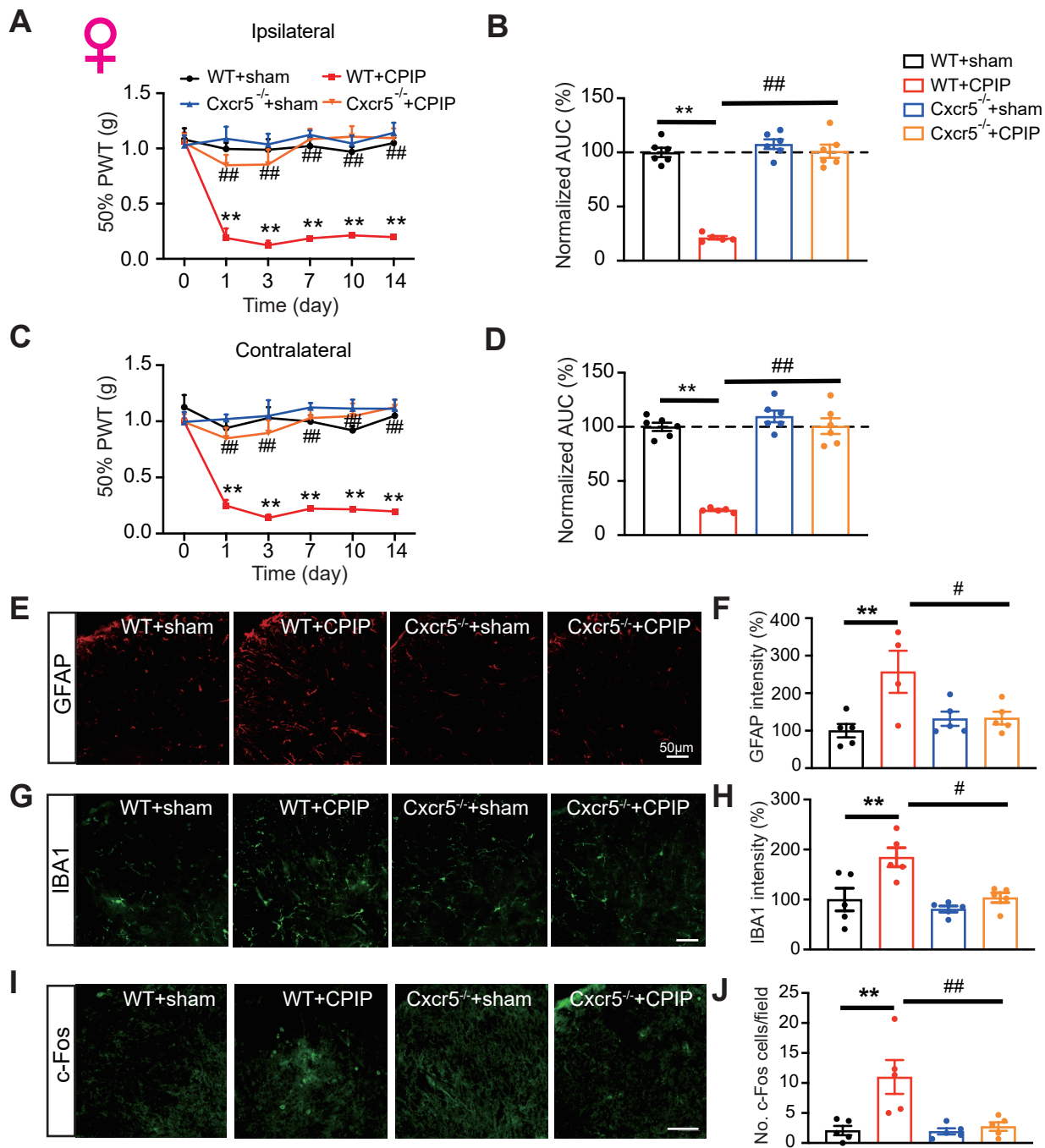

Supplement: Supplementary file 8 — Additional file 8: Figure S8. CXCR5 is important for mechanical allodynia as well as c-Fos and glial cell overactivation in ipsilateral spinal cord dorsal horn of female CPIP model mice.Time course showing 50% PWT changes in ipsilateral hindpaws of WT and Cxcr5−/− female mice after model establishment.Summary of normalized AUC of curves in panel A.Time course showing 50% PWT changes in contralateral hindpaws of WT and Cxcr5−/− female mice after model establishment.Summary of normalized AUC of curves in panel C.Immunostaining of GFAP, Iba1and c-Fosin ipsilateral SCDH from WT + sham, WT + CPIP, Cxcr5−/− + sham and Cxcr5−/− + CPIP groups of female mice.Summary of GFAP& Iba1fluorescence intensity and c-Fospositively stained cell number. n = 5–6 mice/group. **p < 0.01 vs. WT + sham group; #p < 0.05, ##p < 0.01 vs. WT + CPIP group. Scale bar indicates 50 μm. Two-way ANOVA with repeated measures followed by Tukey’s post hoc test was used for comparisons in panels A&C. One-way ANOVA followed by Tukey’s post hoc test was used for comparisons in others. [file 12974_2023_2778_MOESM8_ESM.pdf]

Suppl. Fig. 9

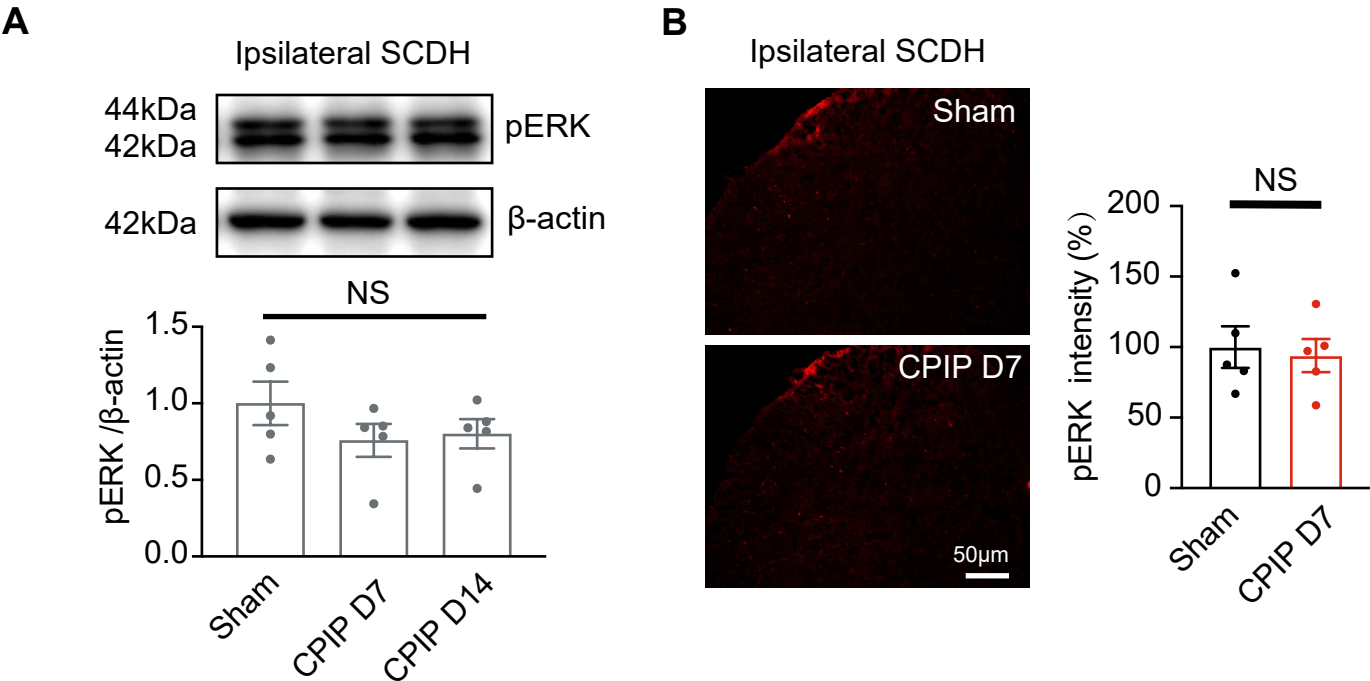

Supplement: Supplementary file 9 — Additional file 9: Figure S9. ERK was not activated in ipsilateral SCDH of CPIP mice.Western blot showing the expression of p-ERK in ipsilateral SCDH of sham and CPIP mice. Upper panels indicated the representative blot images, and lower panel indicate the pooled data.Immunostaining of p-ERK in ipsilateral SCDH of sham and CPIP mice on day 7. n = 5 mice/group. One-way ANOVA followed by Tukey’s post hoc test was used in panel A. Student’s t test was used in panel B. [file 12974_2023_2778_MOESM9_ESM.pdf]
