# Supplementary material for: CXCL13 contributes to chronic pain of a mouse model of CRPS-I via CXCR5-mediated NF-κB activation and pro-inflammatory cytokine production in spinal cord dorsal horn
Source: J Neuroinflammation. 2023 May 8;20:109. doi: 10.1186/s12974-023-02778-x (PMC10165831; doi:10.1186/s12974-023-02778-x)
Supplement: Supplementary file 10 — Additional file 10: Table S1. Sequences of primers used for qPCR. [file 12974_2023_2778_MOESM10_ESM.pdf]

Figure 1 E

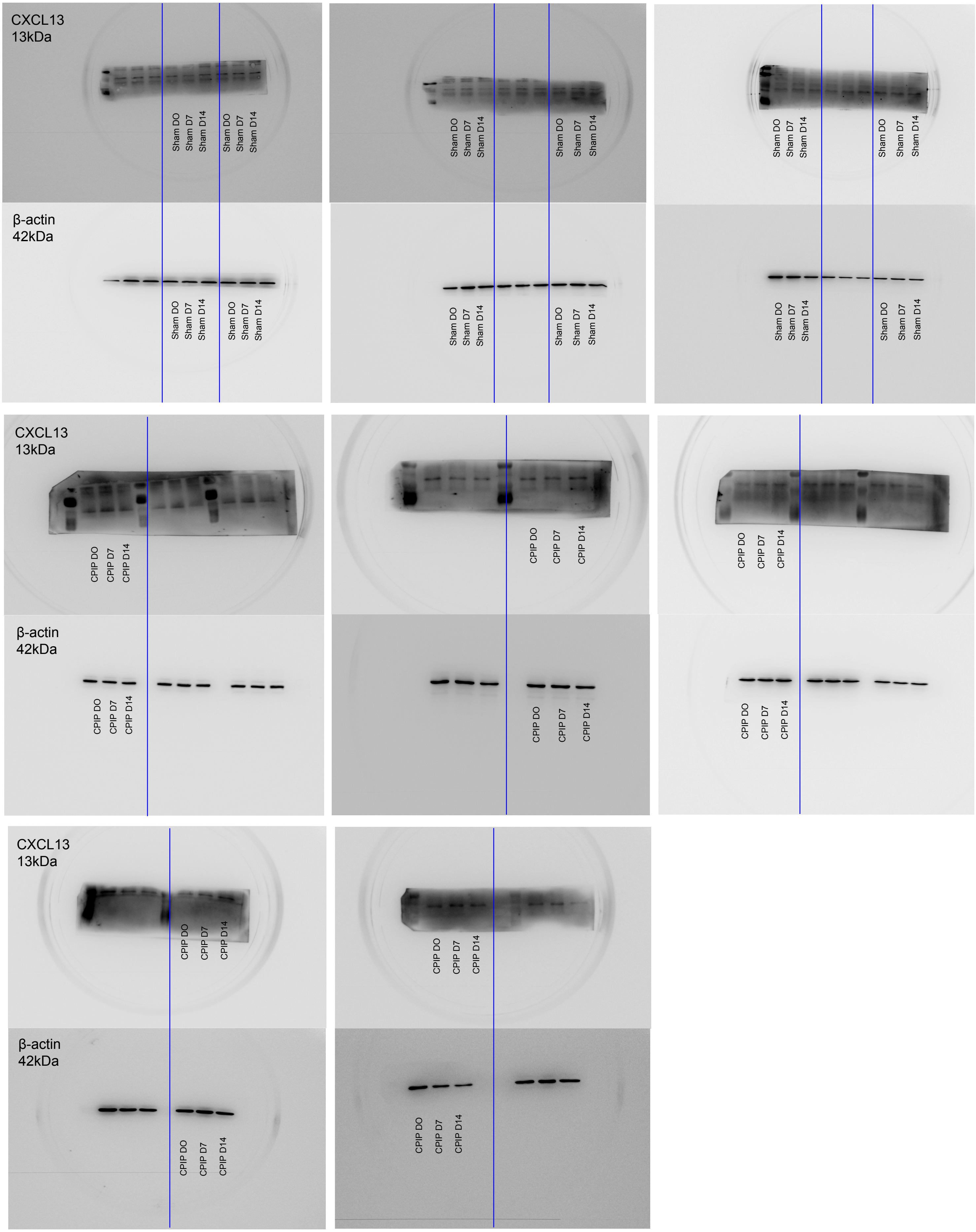
CXCL13
13kDa
Sham DO
Sham D7
Sham D14
Sham DO
Sham D7
Sham D14
β-actin
42kDa
CPIP DO
CPIP D7
CPIP D14

Figure 2 C

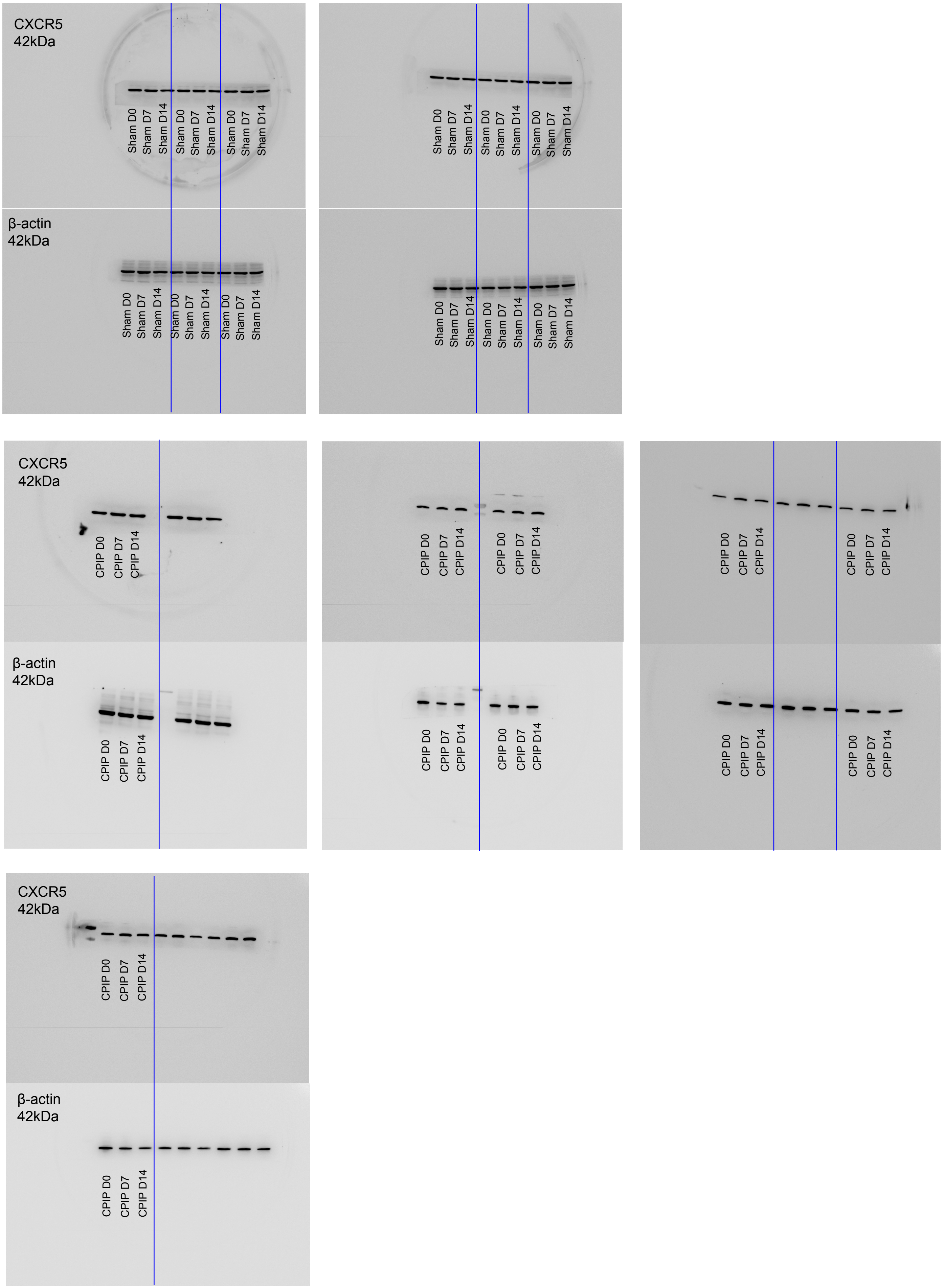
CXCR5
42kDa
β-actin
42kDa
Sham D0
Sham D7
Sham D14
CPIP D0
CPIP D7
CPIP D14

Figure 6

A. Ipsilateral SC Sham GROUP

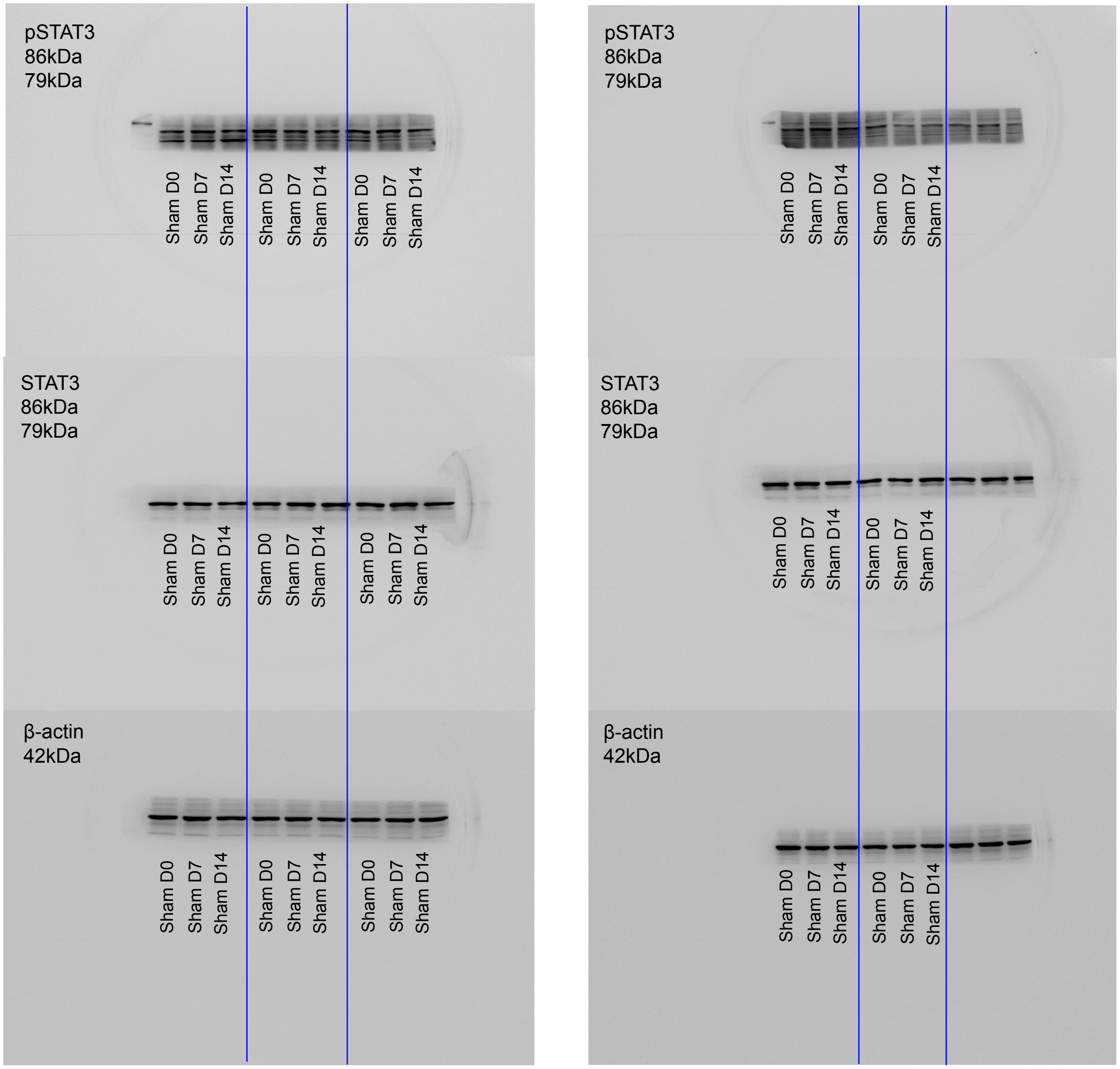

Figure 6

A. Ipsilateral SC CPIP GROUP

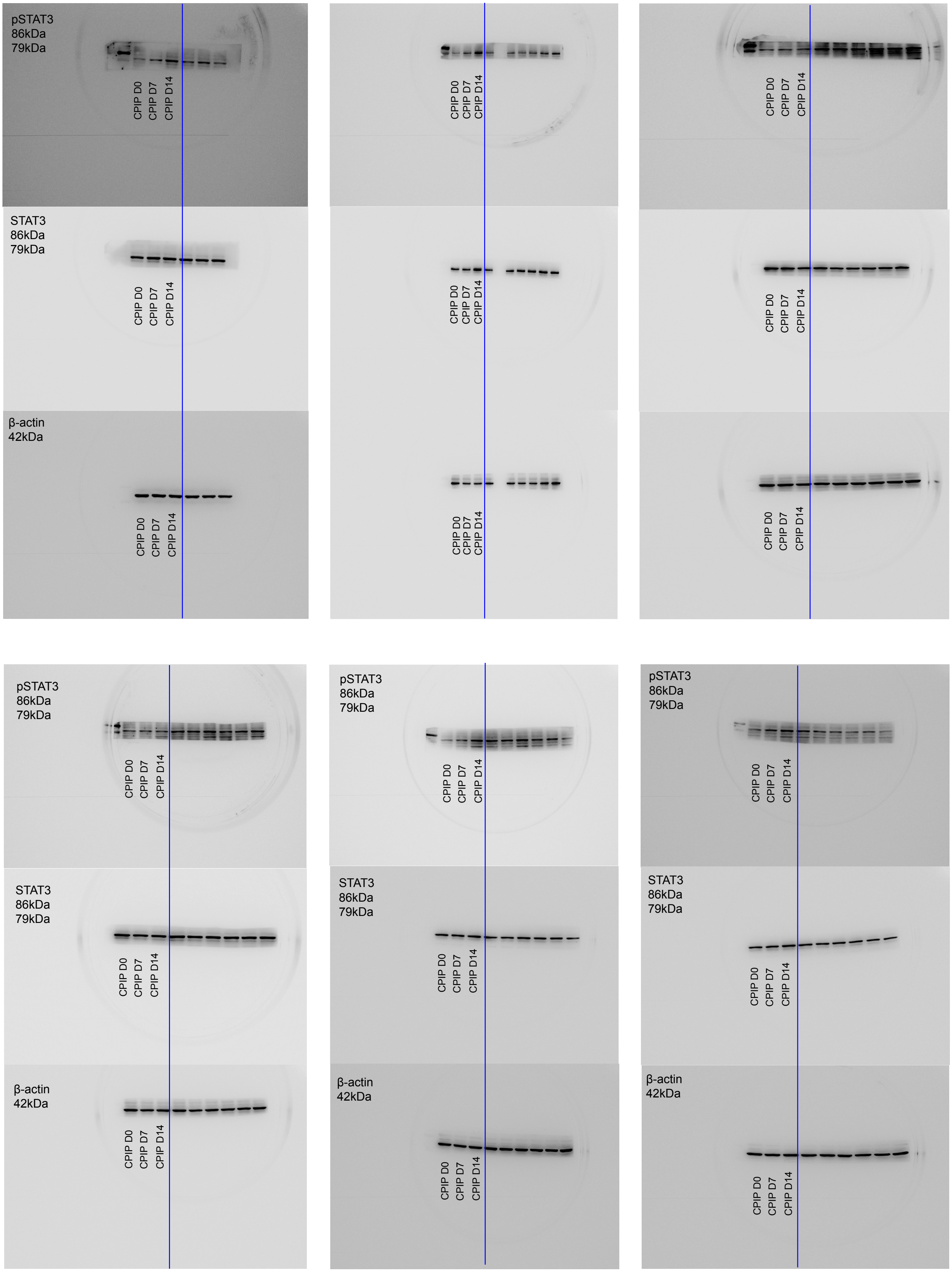

Figure 6 B

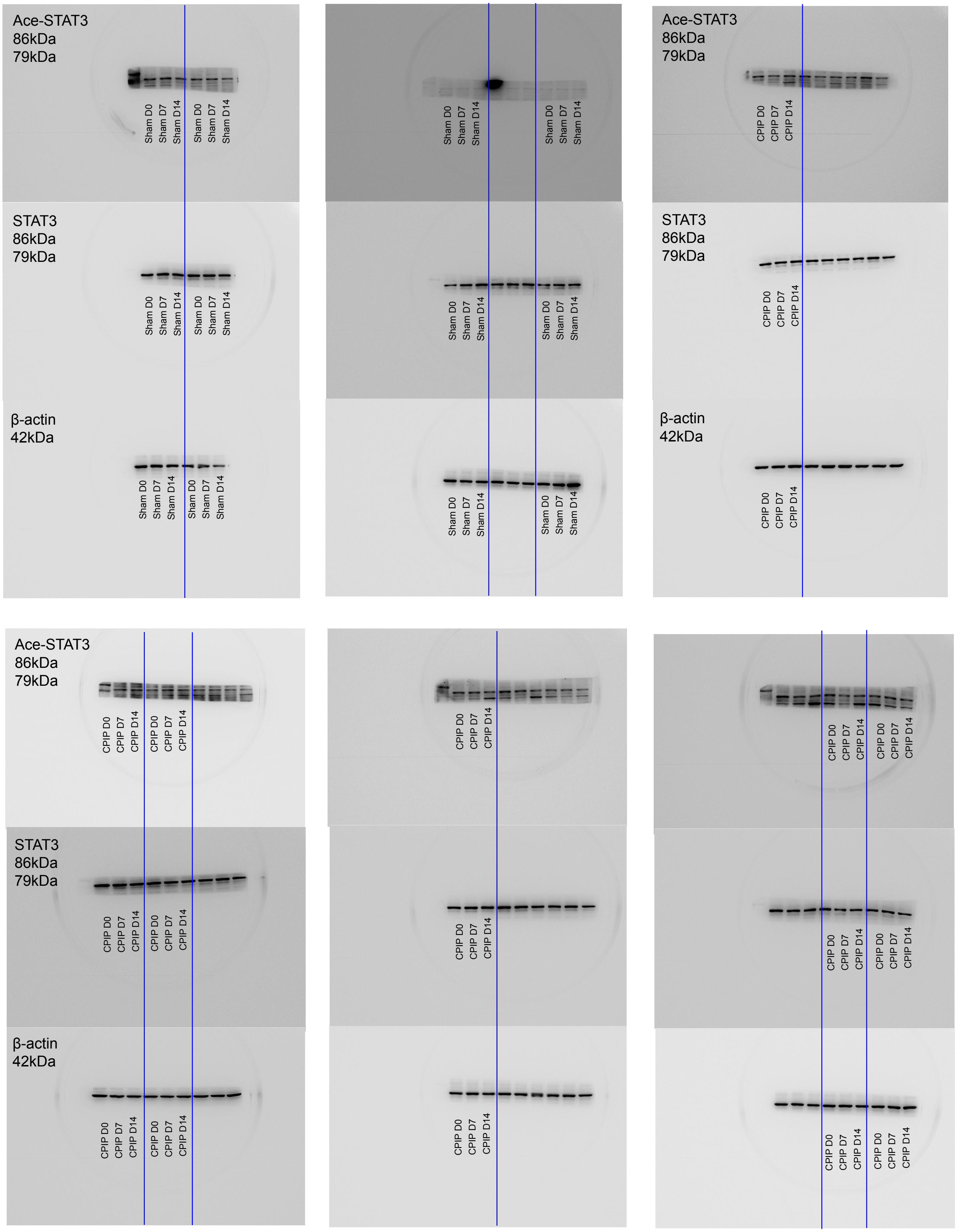
Ace-STAT3
86kDa
79kDa
Sham D0
Sham D7
Sham D14
Sham D0
Sham D7
Sham D14
STAT3
86kDa
79kDa
Sham D0
Sham D7
Sham D14
Sham D0
Sham D7
Sham D14
β-actin
42kDa
Sham D0
Sham D7
Sham D14
Sham D0
Sham D7
Sham D14
Sham D0
Sham D7
Sham D14
Sham D0
Sham D7
Sham D14
Sham D0
Sham D7
Sham D14
Sham D0
Sham D7
Sham D14
Sham D0
Sham D7
Sham D14
Sham D0
Sham D7
Sham D14
Ace-STAT3
86kDa
79kDa
CPIP D0
CPIP D7
CPIP D14
STAT3
86kDa
79kDa
CPIP D0
CPIP D7
CPIP D14
β-actin
42kDa
CPIP D0
CPIP D7
CPIP D14
Ace-STAT3
86kDa
79kDa
CPIP D0
CPIP D7
CPIP D14
CPIP D0
CPIP D7
CPIP D14
STAT3
86kDa
79kDa
CPIP D0
CPIP D7
CPIP D14
CPIP D0
CPIP D7
CPIP D14
β-actin
42kDa
CPIP D0
CPIP D7
CPIP D14
CPIP D0
CPIP D7
CPIP D14
CPIP D0
CPIP D7
CPIP D14
CPIP D0
CPIP D7
CPIP D14
CPIP D0
CPIP D7
CPIP D14
CPIP D0
CPIP D7
CPIP D14
CPIP D0
CPIP D7
CPIP D14
CPIP D0
CPIP D7
CPIP D14
CPIP D0
CPIP D7
CPIP D14
CPIP D0
CPIP D7
CPIP D14

Figure 7 B

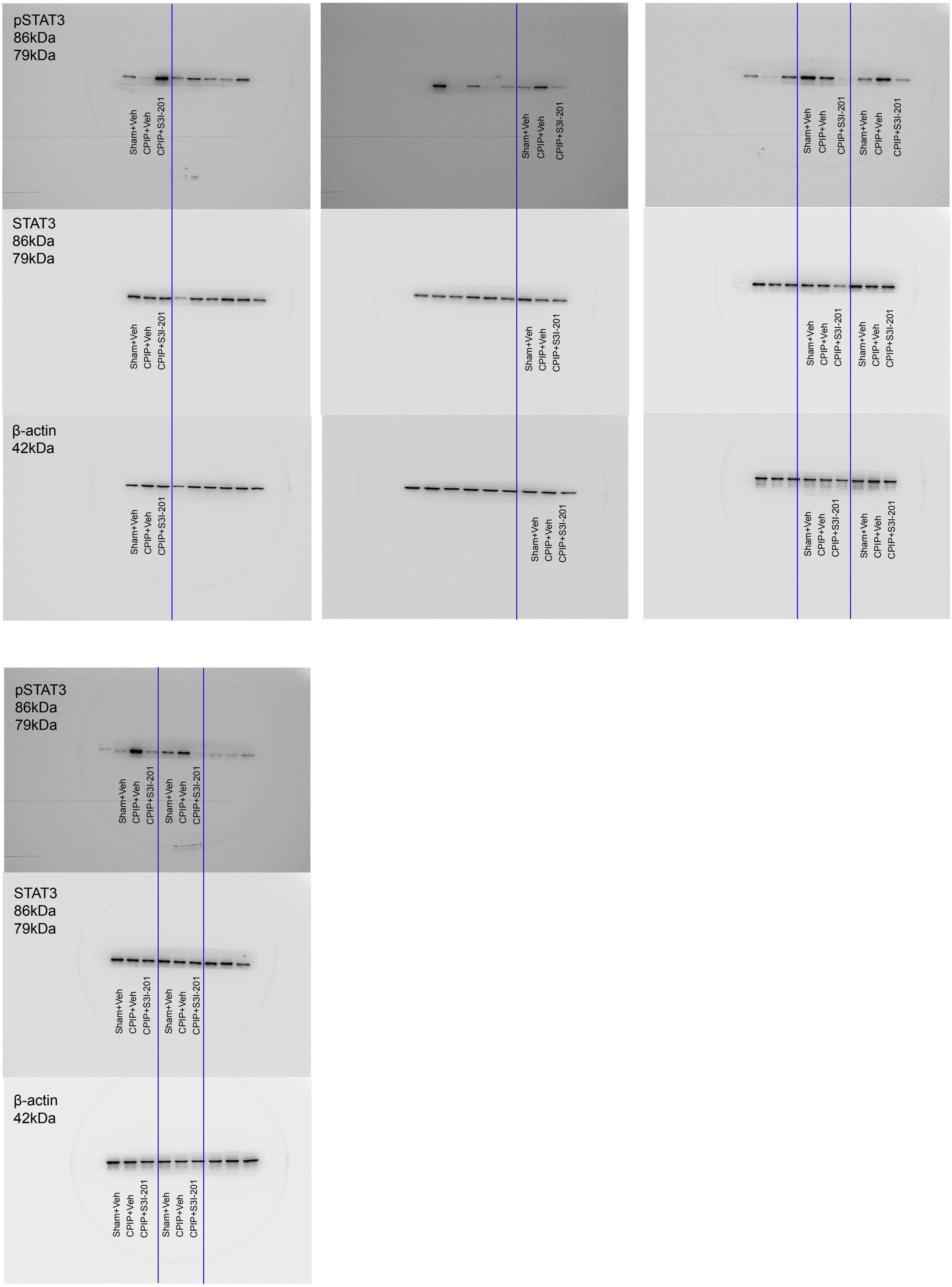
pSTAT3
86kDa
79kDa
Sham+Veh
CPIP+Veh
CPIP+S3I-201
STAT3
86kDa
79kDa
β-actin
42kDa

Figure 7 C

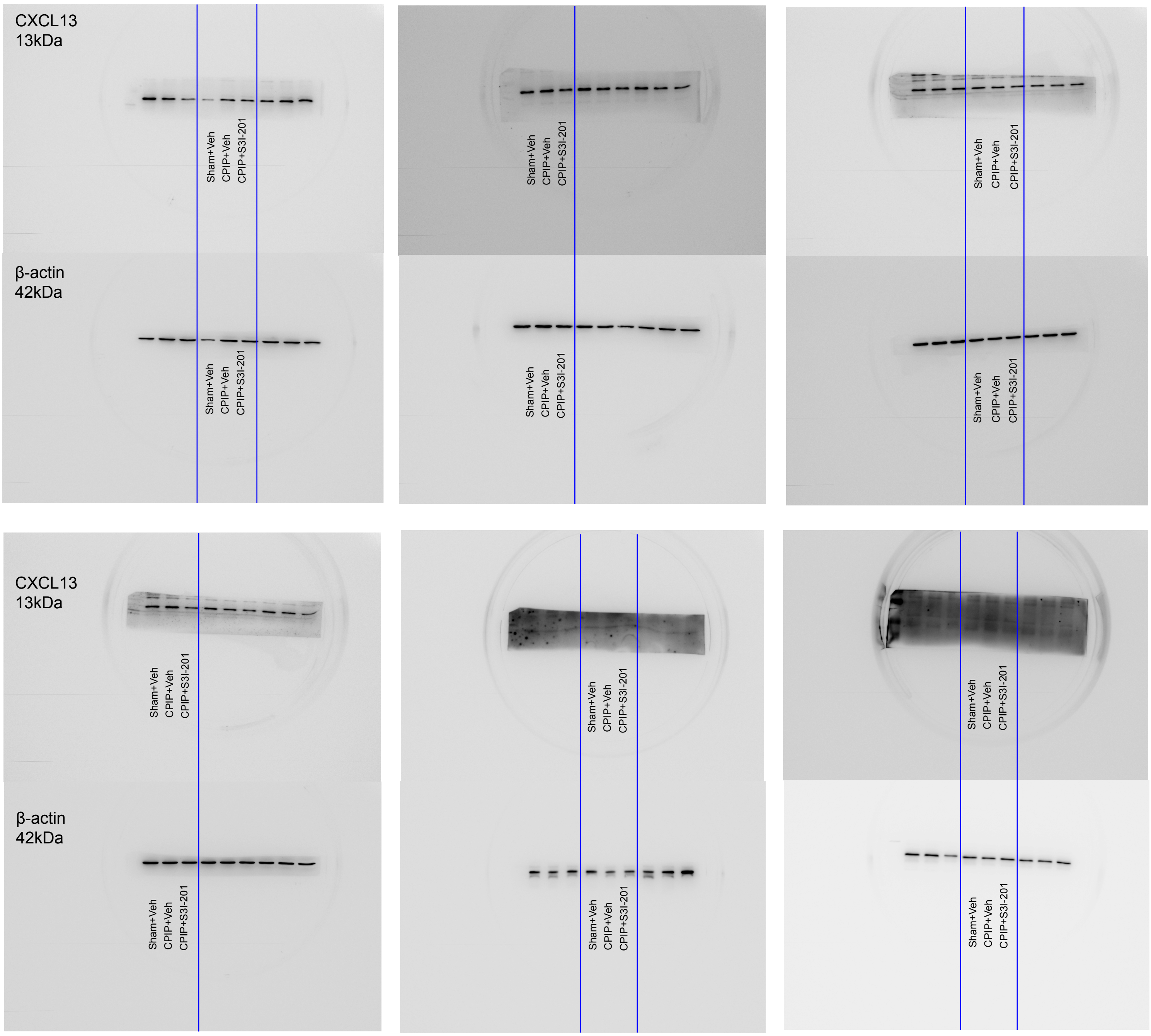
CXCL13
13kDa
Sham+Veh
CPIP+Veh
CPIP+S3I-201
Sham+Veh
CPIP+Veh
CPIP+S3I-201
Sham+Veh
CPIP+Veh
CPIP+S3I-201
β-actin
42kDa
Sham+Veh
CPIP+Veh
CPIP+S3I-201
Sham+Veh
CPIP+Veh
CPIP+S3I-201
Sham+Veh
CPIP+Veh
CPIP+S3I-201
CXCL13
13kDa
Sham+Veh
CPIP+Veh
CPIP+S3I-201
Sham+Veh
CPIP+Veh
CPIP+S3I-201
Sham+Veh
CPIP+Veh
CPIP+S3I-201
β-actin
42kDa
Sham+Veh
CPIP+Veh
CPIP+S3I-201
Sham+Veh
CPIP+Veh
CPIP+S3I-201
Sham+Veh
CPIP+Veh
CPIP+S3I-201

Figure 8 A

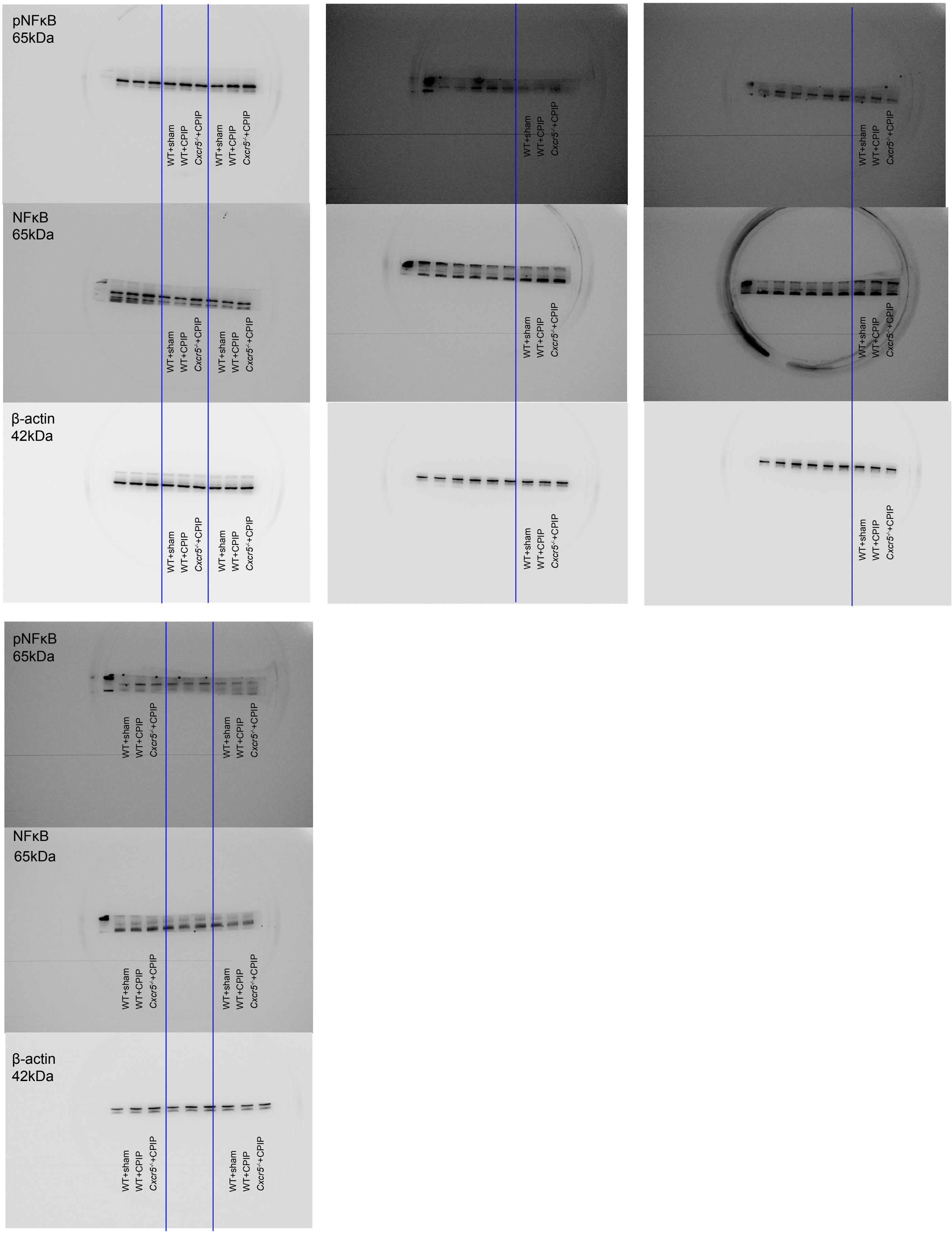
pNFκB
65kDa
NFκB
65kDa
β-actin
42kDa
WT+sham
WT+CPIP
Cxcr5-/-+CPIP

Fig 9 A

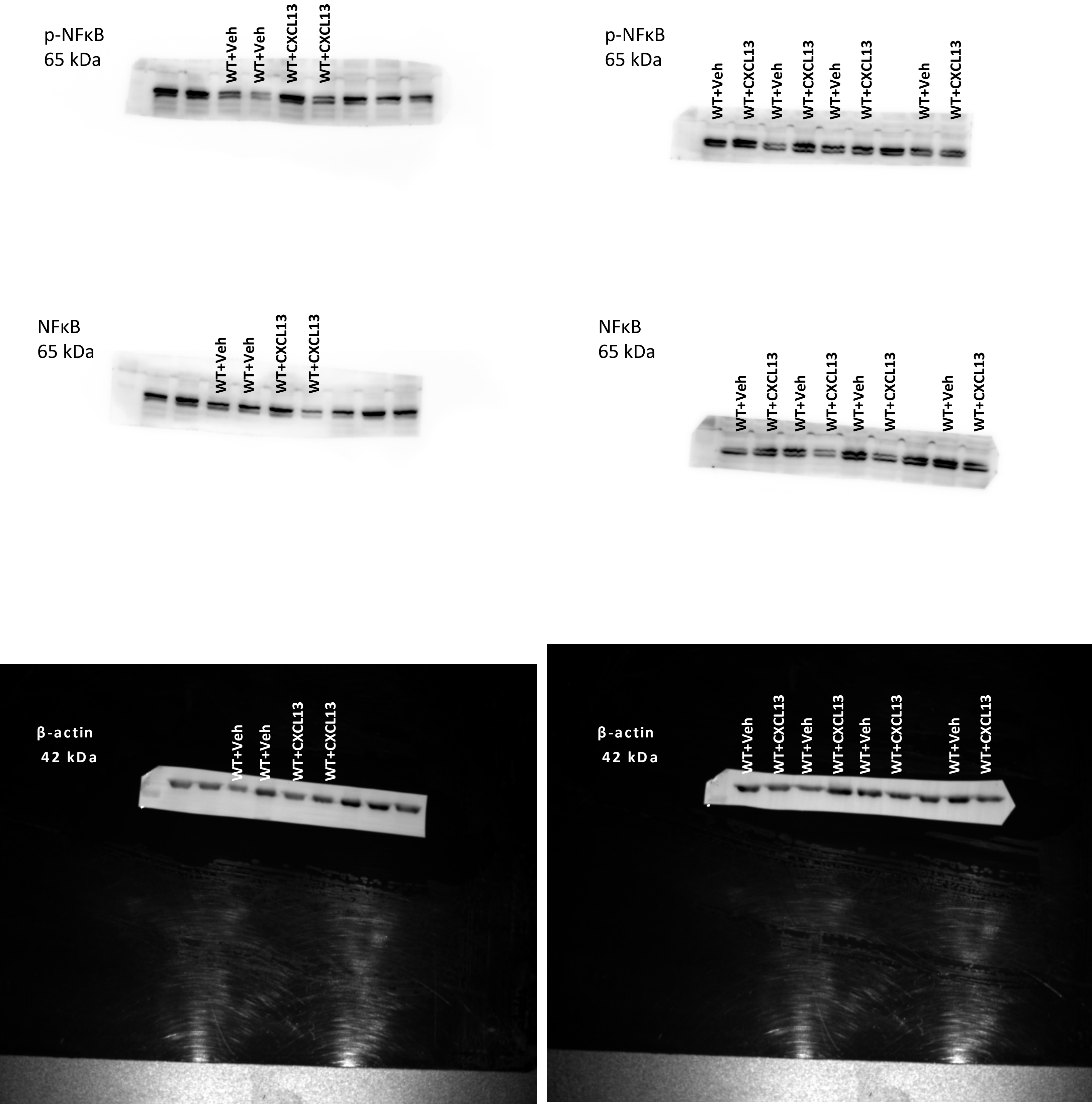
p-NFκB
65 kDa
WT+Veh
WT+Veh
WT+CXCL13
WT+CXCL13
p-NFκB
65 kDa
WT+Veh
WT+CXCL13
WT+Veh
WT+CXCL13
WT+Veh
WT+CXCL13
WT+Veh
WT+CXCL13
NFκB
65 kDa
WT+Veh
WT+Veh
WT+CXCL13
WT+CXCL13
NFκB
65 kDa
WT+Veh
WT+CXCL13
WT+Veh
WT+CXCL13
WT+Veh
WT+CXCL13
WT+Veh
WT+CXCL13
β-actin
42 kDa
WT+Veh
WT+Veh
WT+CXCL13
WT+CXCL13
β-actin
42 kDa
WT+Veh
WT+CXCL13
WT+Veh
WT+CXCL13
WT+Veh
WT+CXCL13
WT+Veh
WT+CXCL13

Fig 9 C

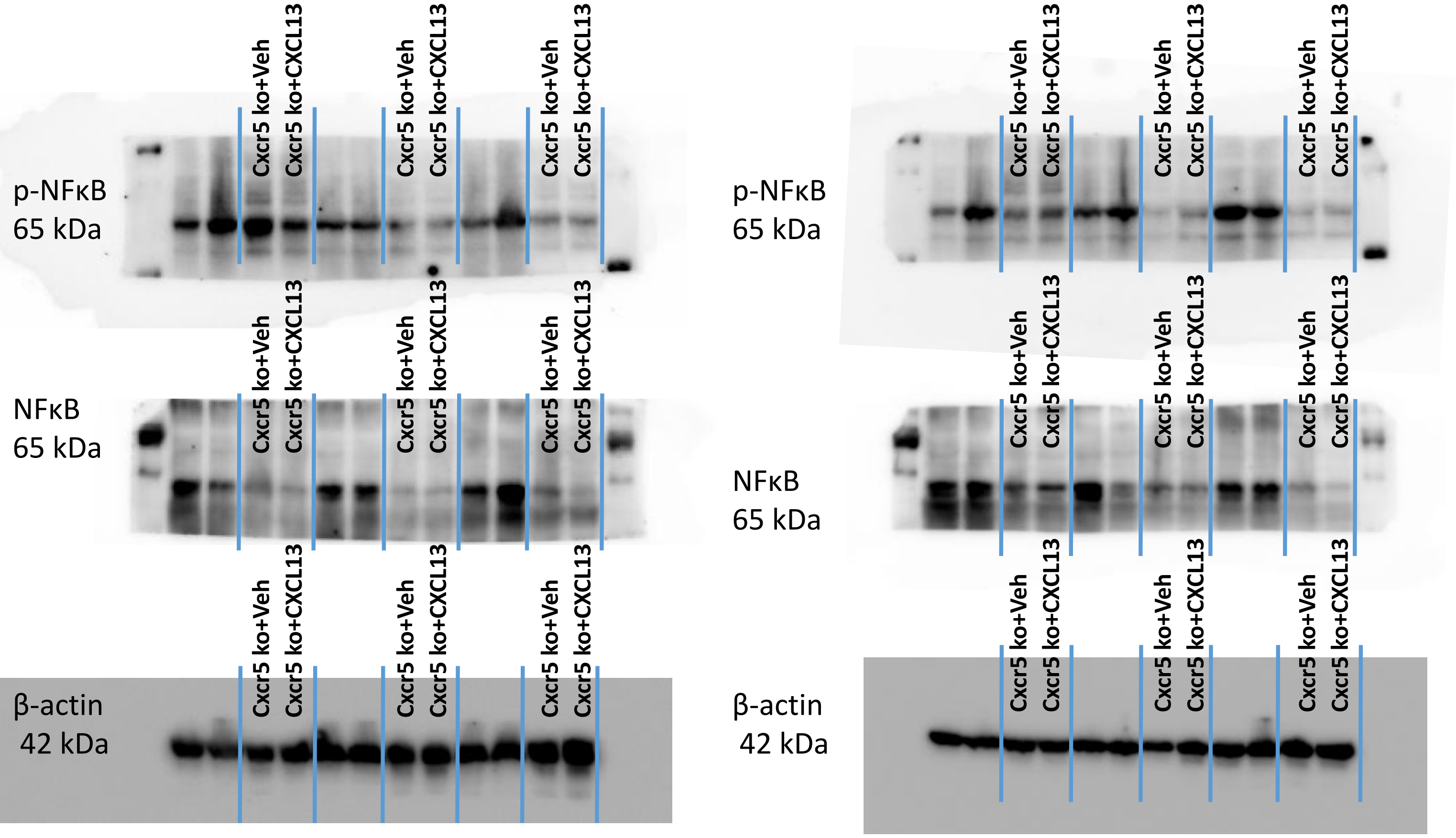
p-NFκB
65 kDa
NFκB
65 kDa
β-actin
42 kDa
Cxcr5 ko+Veh
Cxcr5 ko+CXCL13

Fig 10 D

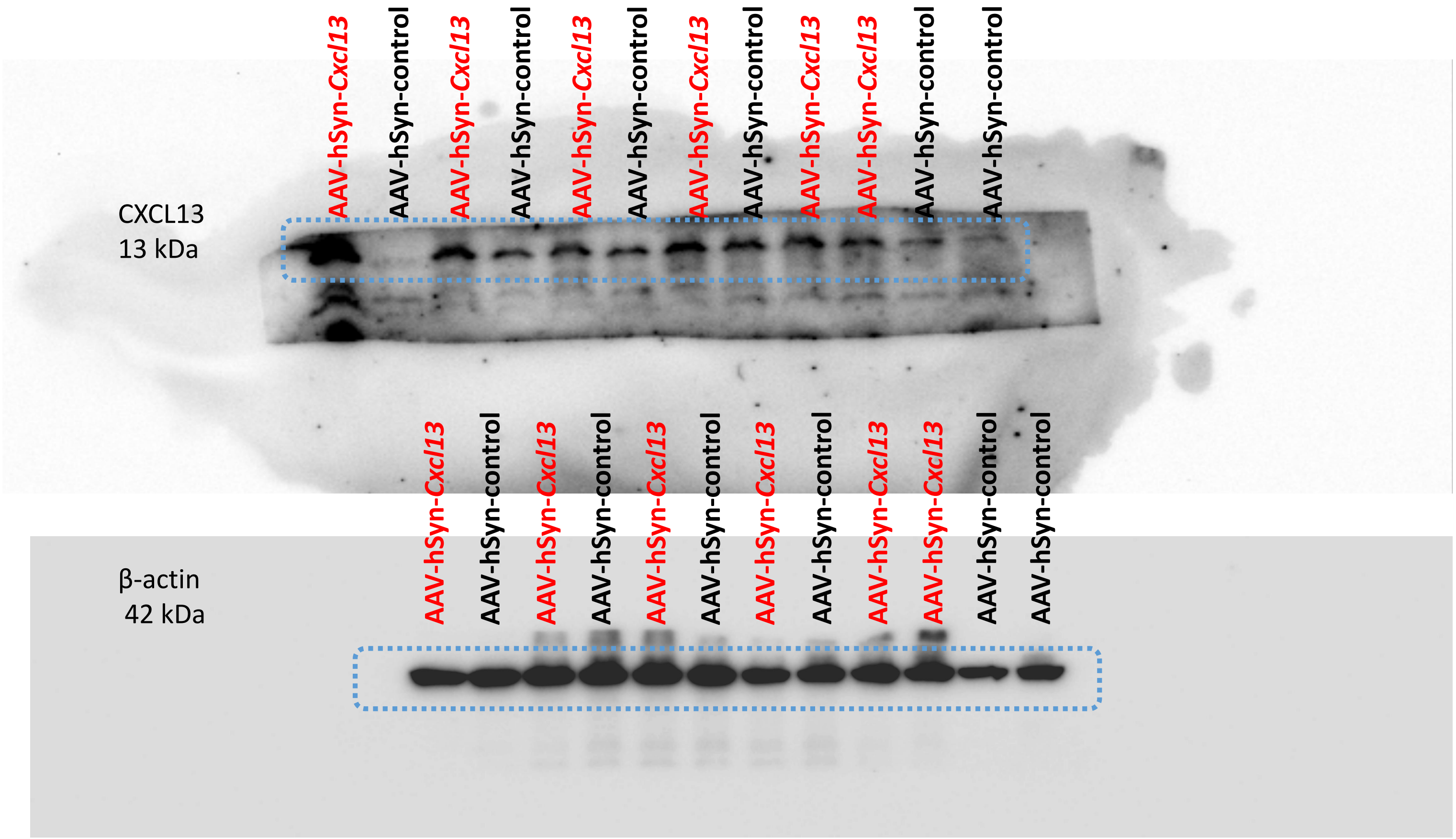
AAV-hSyn-*Cxcl13*
AAV-hSyn-control
AAV-hSyn-*Cxcl13*
AAV-hSyn-control
AAV-hSyn-*Cxcl13*
AAV-hSyn-control
AAV-hSyn-*Cxcl13*
AAV-hSyn-control
AAV-hSyn-*Cxcl13*
AAV-hSyn-*Cxcl13*
AAV-hSyn-control
AAV-hSyn-control
CXCL13
13 kDa
AAV-hSyn-*Cxcl13*
AAV-hSyn-control
AAV-hSyn-*Cxcl13*
AAV-hSyn-control
AAV-hSyn-*Cxcl13*
AAV-hSyn-control
AAV-hSyn-*Cxcl13*
AAV-hSyn-control
AAV-hSyn-*Cxcl13*
AAV-hSyn-*Cxcl13*
AAV-hSyn-control
AAV-hSyn-control
β-actin
42 kDa

Spl. Fig. 2

A

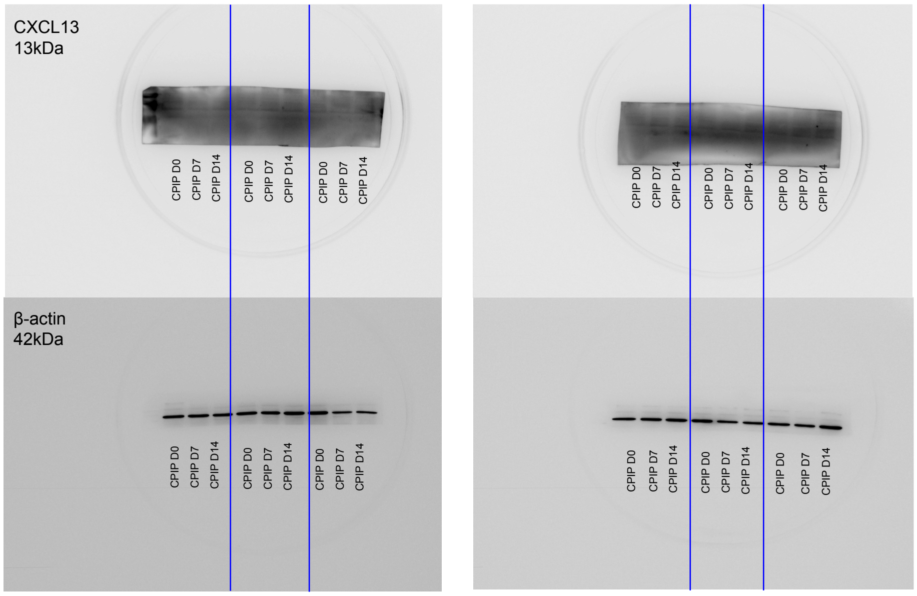

CXCL13
13kDa
β-actin
42kDa
CPIP D0
CPIP D7
CPIP D14

B

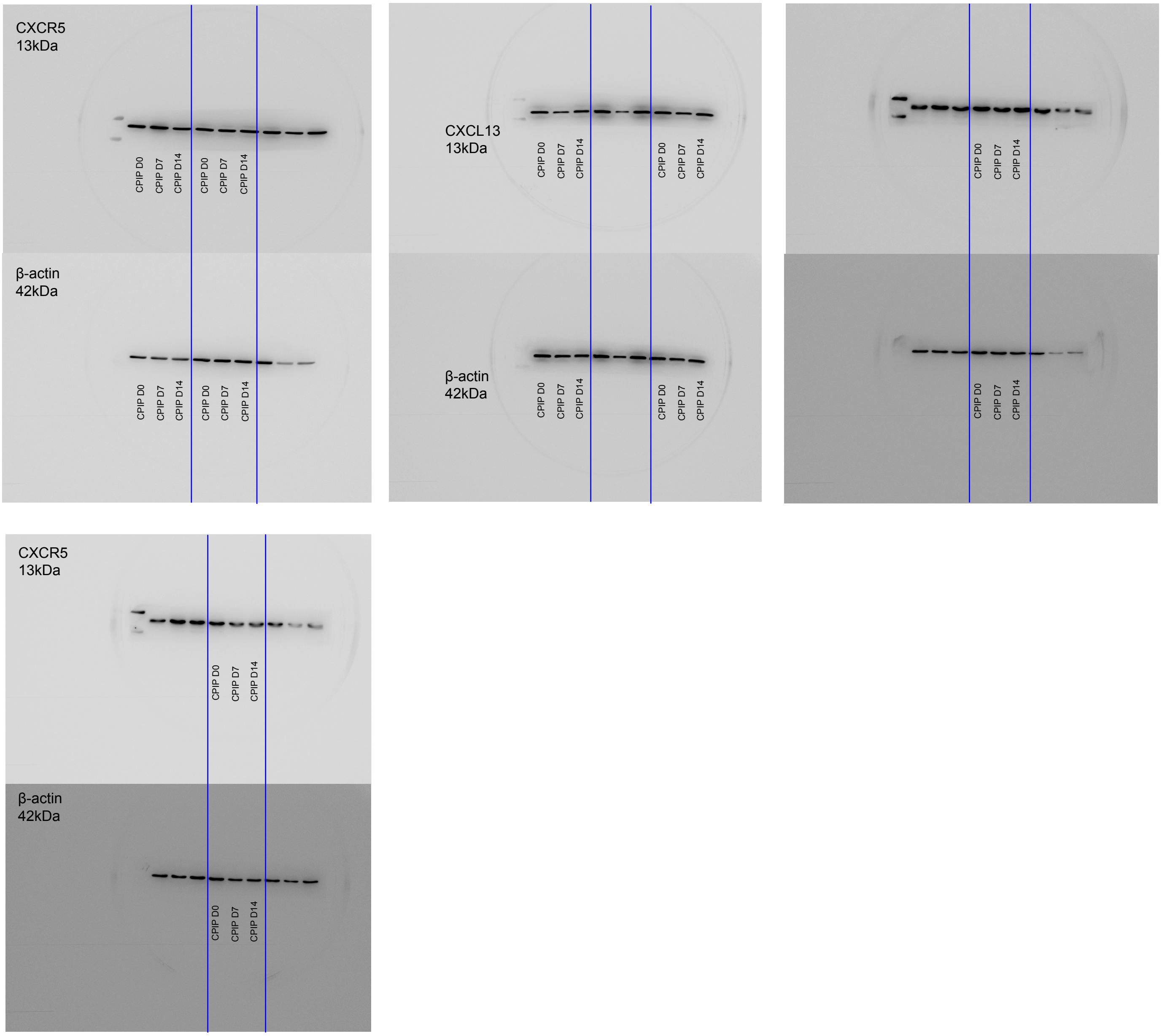

CXCR5
13kDa
β-actin
42kDa
CXCL13
13kDa
CPIP D0
CPIP D7
CPIP D14

Spl.Fig.4 A

Ipsilateral L3-L5 DRG

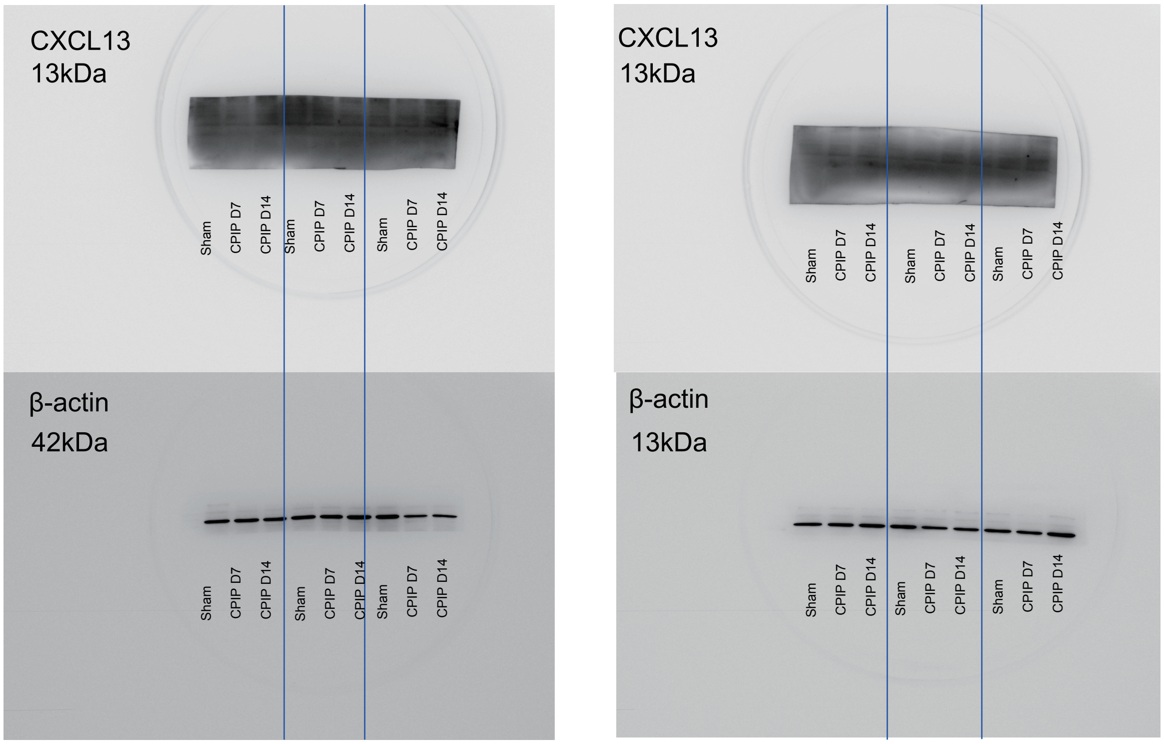

Spl.Fig.4 B

Ipsilateral L3-L5 DRG

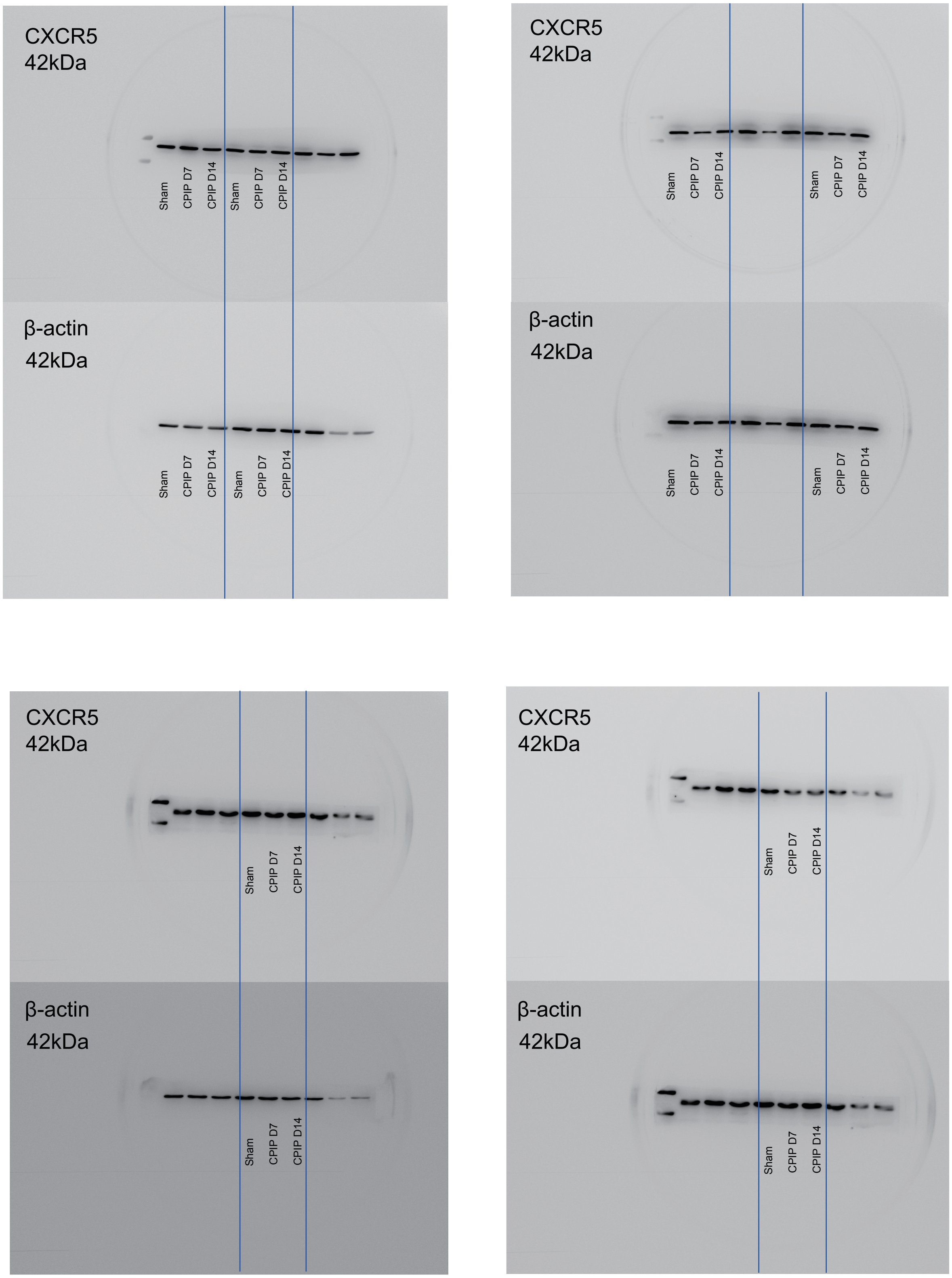

Spl. Fig. 9A

A

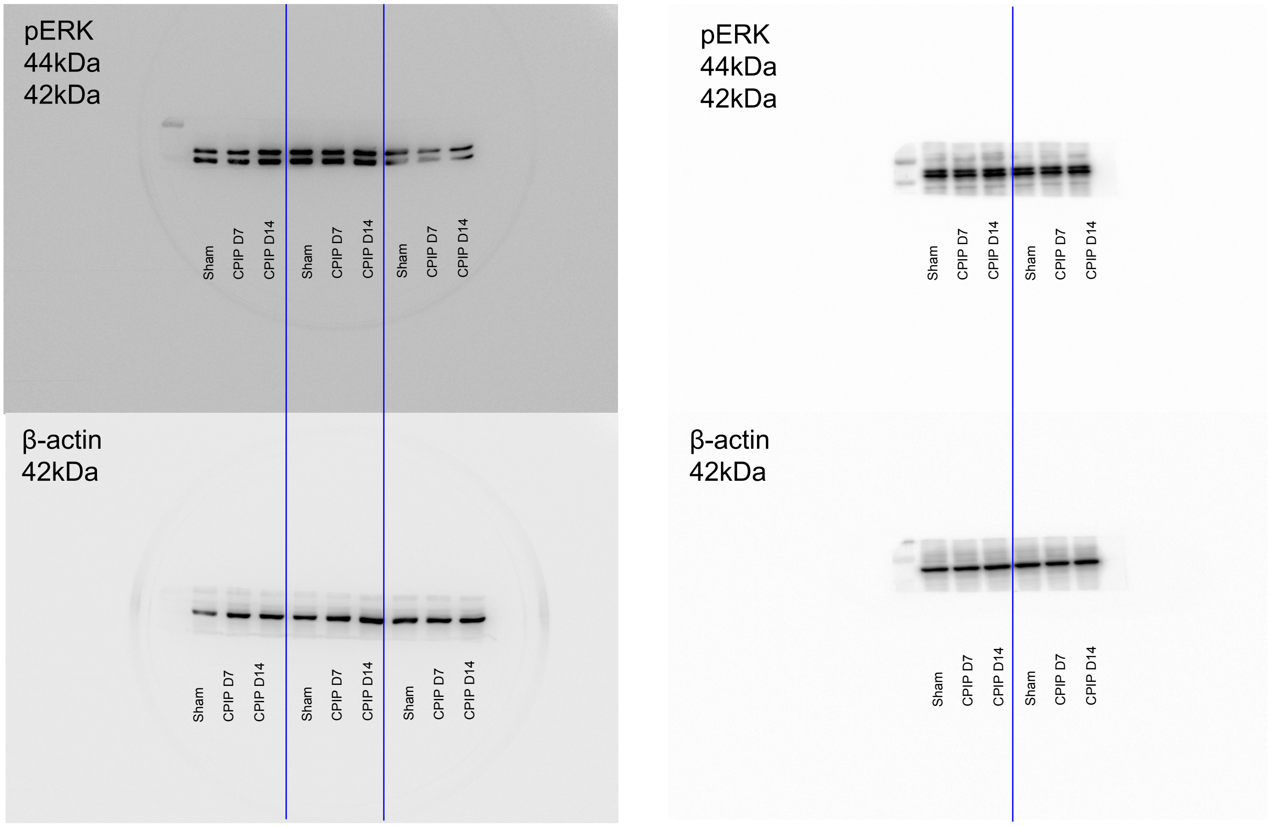
pERK
44kDa
42kDa
Sham
CPIP D7
CPIP D14
Sham
CPIP D7
CPIP D14
Sham
CPIP D7
CPIP D14
pERK
44kDa
42kDa
Sham
CPIP D7
CPIP D14
Sham
CPIP D7
CPIP D14
β-actin
42kDa
Sham
CPIP D7
CPIP D14
Sham
CPIP D7
CPIP D14
Sham
CPIP D7
CPIP D14
β-actin
42kDa
Sham
CPIP D7
CPIP D14
Sham
CPIP D7
CPIP D14
