# Supplementary material for: CXCL13 contributes to chronic pain of a mouse model of CRPS-I via CXCR5-mediated NF-κB activation and pro-inflammatory cytokine production in spinal cord dorsal horn
Source: J Neuroinflammation. 2023 May 8;20:109. doi: 10.1186/s12974-023-02778-x (PMC10165831; doi:10.1186/s12974-023-02778-x)
Supplement: Supplementary file 3 [file 12974_2023_2778_MOESM11_ESM.docx]

Suppl. Table 1 Sequences of the primers

Gene name	Gene ID	Primer sequence（5’to 3’）	A
β-actin	11461	F: 5’-GTGCTATGTTGCTCTAGACTTCG-3’	17
		R: 5’-ATGCCACAGGATTCCATACC-3’	
Cxcl13	55985	F: 5’-TTGTGATCTGGACCAAGATGAA-3’	10
		R: 5’-GACTTTTGCTTTGGACATGTCT-3’	
Cxcr5	12145	F: 5’-GAAAACGAAGCGGAAACTAGAG-3’	22
		R: 5’-AGGAAGATGACAATGTGGTAGG-3’	
Il-6	16193	F: 5’-CTCCCAACAGACCTGTCTATAC-3’	97
		R: 5’-CCATTGCACAACTCTTTTCTCA-3’	
Ccl2	20296	F: 5’-TTAAAAACCTGGATCGGAACCAA-3’	12
		R: 5’-GCATTAGCTTCAGATTTACGGGT-3’	
Ccl3	20302	F: 5’-TTCTCTGTACCATGACACTCTGC-3’	10
		R: 5’-CGTGGAATCTTCCGGCTGTAG-3’	
